# Label‐Free Single‐Molecule Immunoassay

**DOI:** 10.1002/advs.202505207

**Published:** 2025-06-20

**Authors:** Xiaoyan Zhou, Chao Chen, Shuang Zhou, Guangzhong Ma, Mohammad Javad H. N. Chemerkouh, Christine L. H. Snozek, Eric H. Yang, Jiapei Jiang, Brandyn Braswell, Zijian Wan, Xinyu Zhou, Shaopeng Wang

**Affiliations:** ^1^ Center for Bioelectronics and Biosensors The Biodesign Institute Arizona State University Tempe AZ 85287 USA; ^2^ School of Electrical Computer and Energy Engineering Arizona State University Tempe AZ 85287 USA; ^3^ School of Biological and Health Systems Engineering Arizona State University Tempe AZ 85287 USA; ^4^ School of Mathematical and Statistical Sciences Arizona State University Tempe AZ 85287 USA; ^5^ Department of Laboratory Medicine and Pathology Mayo Clinic Arizona Phoenix AZ 85054 USA; ^6^ Department of Cardiovascular Disease Mayo Clinic Arizona Phoenix AZ 85054 USA; ^7^ School of Engineering for Matter Transport and Energy Arizona State University Tempe AZ 85287 USA

**Keywords:** digital immunoassay, label‐free, plasmonic scattering microscopy, single‐molecule, whole blood

## Abstract

Single‐molecule immunoassay is a reliable technique for the detection and quantification of low‐abundance blood biomarkers, which are essential for early disease diagnosis and biomedical research. However, current single‐molecule methods predominantly rely on endpoint detection and necessitate signal amplification via labeling, which brings a variety of unwanted effects, like matrix effect and autofluorescence interference. This study introduces a real‐time mass imaging‐based label‐free single‐molecule immunoassay (LFSMiA). Featuring plasmonic scattering microscopy‐based mass imaging, a 2‐step sandwich assay format enables background reduction, minimization of matrix effect by dynamic tracking of single binding events, and fully leveraging real‐time data for improved measurement precision through a Bayesian Gaussian process model, the LFSMiA enables ultra‐sensitive and direct protein detection at the single‐molecule level in neat blood sample matrices. LFSMiA measurement is demonstrated for interleukin‐6 and prostate‐specific antigen in buffer, undiluted serum, and whole blood with sub‐femtomolar detection limits and eight logs of dynamic ranges. Moreover, comparable performance is achieved with an inexpensive miniaturized setup. To show its translational potential to clinical settings and point‐of‐care diagnostics, N‐terminal pro‐B‐type natriuretic peptide is examined in patient whole blood samples using the LFSMiA and results in a strong linear correlation (r > 0.99) with standard clinical lab results.

## Graphical abstract

Label‐free single‐molecule immunoassay (LFSMiA) enables ultra‐sensitive and wide dynamic range protein detection in whole blood samples. LFSMiA features real‐time single‐molecule imaging by plasmonic scattering microscopy, background reduction by a 2‐step sandwich assay, minimizing matrix effect by dynamic tracking of single binding events, and improving measurement precision by fully leveraging real‐time data through a Bayesian Gaussian process model.

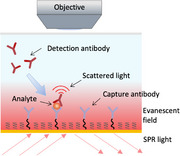

## 1 Introduction

Detection and quantification of biomarkers at extremely low levels are important to disease detection, monitoring, and treatment.^[^ ^1^ ^]^ While numerous approaches have been developed, the enzyme‐linked immunosorbent assay remains the “gold standard” method for the detection of many protein biomarkers in both clinical use and basic research.^[^ ^2^ ^]^ However, its dynamic range (log 3), detection limit (picomolar range), and assay time (3–8 h) fall short of the requirement of early disease diagnosis in clinical settings.^[^ ^3^ ^]^ Recently, the development of single‐molecule imaging has made it possible to visualize individual binding complexes, leading to greatly improved sensitivity, reliability, and more depth of information compared to conventional methods.^[^ ^4^ ^]^ Newer methods – including single‐molecule enzyme‐linked immunosorbent assays (SiMoAs),^[^ ^5^ ^]^ single‐molecule recognition through equilibrium Poisson sampling,^[^ ^6^ ^]^ single‐molecule augmented capture,^[^ ^7^ ^]^ and time‐resolved digital immunoassay^[^ ^8^ ^]^ have been developed to improve detection limit. However, these methods must amplify the single molecule binding signal by enzymatic amplification, fluorescence, or nanoparticle labeling. These signal amplification techniques suffer from inherent limitations. Methods based on enzyme amplification and gold nanoparticle labeling are prone to matrix effects and unwanted background signals arising from nonspecific reagent binding to assay substrate. Fluorescence‐based methods are prone to photobleaching and ubiquitous autofluorescence of sample matrix components. These limitations make these single‐molecule assays incapable of measuring biomarkers directly in undiluted complex sample matrices, especially in whole blood. In addition, these assays predominantly rely on end‐point detection and fail to exploit real‐time kinetic data. Since most interactions on the sensor surface follow first‐order binding kinetics and replicate measurements follow a Poisson distribution, real‐time data can contribute meaningfully to the final signal. Therefore, fully leveraging real‐time kinetic information can significantly enhance the precision and reliability of the final results.

Here, we introduce a real‐time label‐free single‐molecule immunoassay (LFSMiA) based on mass imaging, which enables ultra‐sensitive and direct protein detection in neat blood sample matrices. This novel LFSMiA employs plasmonic scattering microscopy (PSM),^[^ ^9^ ^]^ a technique capable of detecting scattered light from single molecules based on their mass property, to monitor the formation of individual biomarker immunocomplexes on the sensor surface. A 2‐step sandwich assay format is adopted in the LFSMiA to separate sample incubation from detection antibody binding. In this way, samples in a complex matrix can be directly incubated with the capture antibody functionalized sensor surface. The sample matrix is washed away at the end of incubation to reduce the background signal, followed by the introduction of a pure detection antibody in the buffer. As such, PSM can image and track the specific binding of individual detection antibody molecules to the captured biomarker molecules on the sensor surface. In addition, the LFSMiA reduces susceptibility to matrix effects, such as interference from molecules structurally like the biomarker or heterophilic antibodies, by dynamically tracking binding events and utilizing binding kinetics data to distinguish between specific and nonspecific interactions. We also use a Bayesian Gaussian process (BGP) model fitting the real‐time binding data to improve detection accuracy. LFSMiA is the first label‐free single molecule immunoassay. The label‐free nature of LFSMiA eliminates complications associated with labeling reagents, which can hinder binding efficiency and lead to nonspecific interactions. To validate this novel method, we detected interleukin‐6 (IL‐6, an important infection and inflammation biomarker) and prostate‐specific antigen (PSA, a widely used prostate cancer biomarker) in both pure buffer and neat serum. We also evaluated the clinical application of LFSMiA with N‐terminal pro‐B‐type natriuretic peptide (NT‐proBNP), an important biomarker for heart failure, and obtained excellent linear correlation with clinical lab results measured with the commercial Elecsys proBNP II assay. To highlight the technology's potential for point‐of‐care diagnostics, we developed an inexpensive and miniaturized version of PSM microscope, which achieved comparable assay performance. Our method is also the first to realize sub‐femtomolar biomarker detection in undiluted whole blood samples. Therefore, we believe that the LFSMiA will be a promising method of revolutionizing in vitro molecular diagnostics.

## 2 Results

### 2.1 Principle of LFSMiA

**Figure 1:**
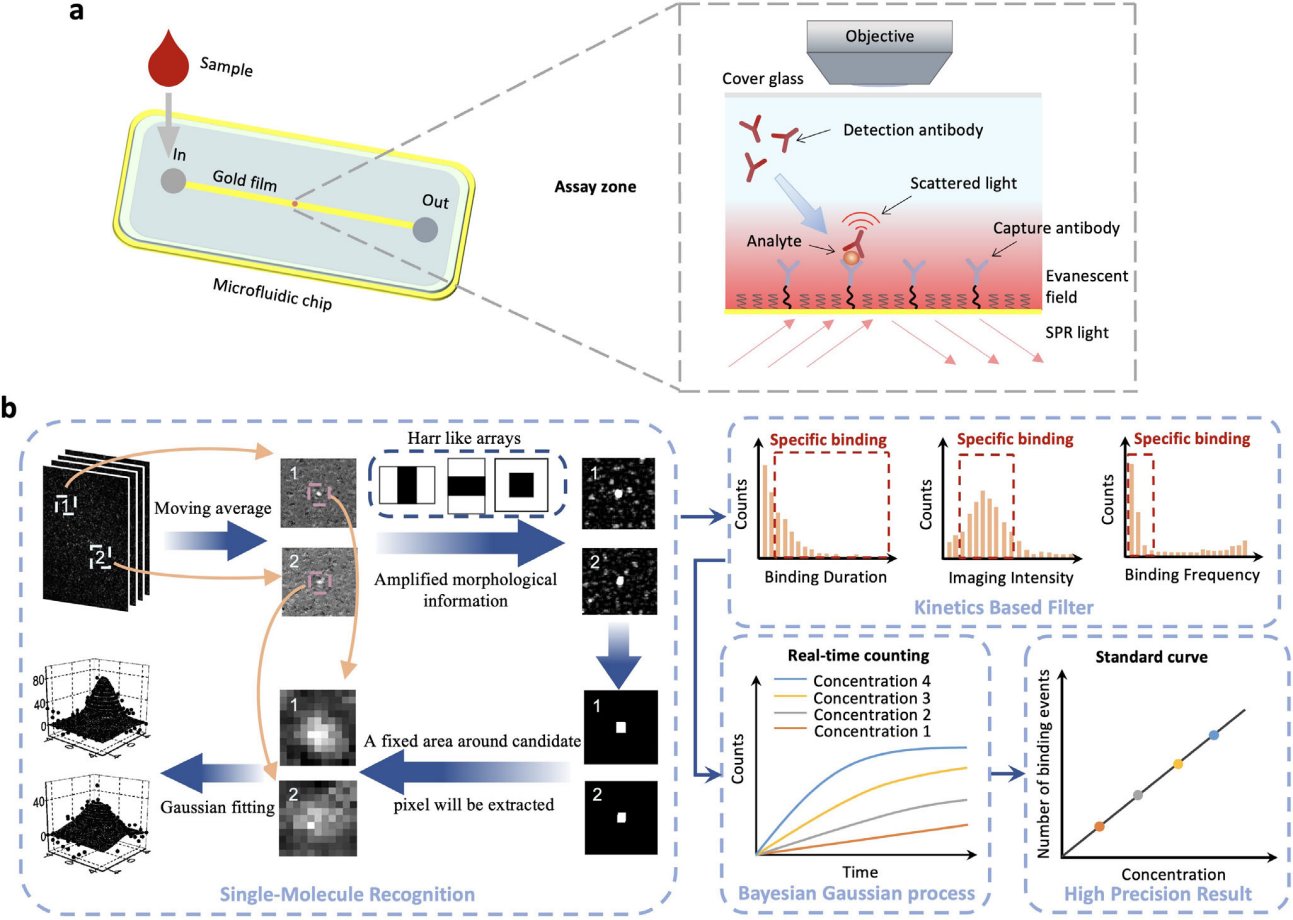
The principle of LFSMiA. a) Experimental setup. The analytes of interest are first pulled down by the capture antibodies tethered to the gold film via an alkane linker. A *p*‐polarized incident light is focused on the gold surface with an incident angle of 71° to excite SPR and the associated evanescent field. After introducing the detection antibody solution, the process of detection antibody binding to the biomarkers caught on the surface is recorded by a CMOS camera via an air objective above the microfluidic channel. b) The raw images are processed by a custom‐written algorithm to remove background noise and to extract the binding events of single particles hitting the sensor surface. A time course of the total count of the specific binding of the detection antibody is then determined and fitted with the Bayesian Gaussian process model to enhance the precision after filtering by the position, molecular weight, binding duration, and binding frequency of the detected binding events. Finally, a standard curve (binding events versus analyte concentration) is generated from triplicate tests of different analyte concentrations. More details are provided in Note S2 (Supporting Information).

**Figure 2:**
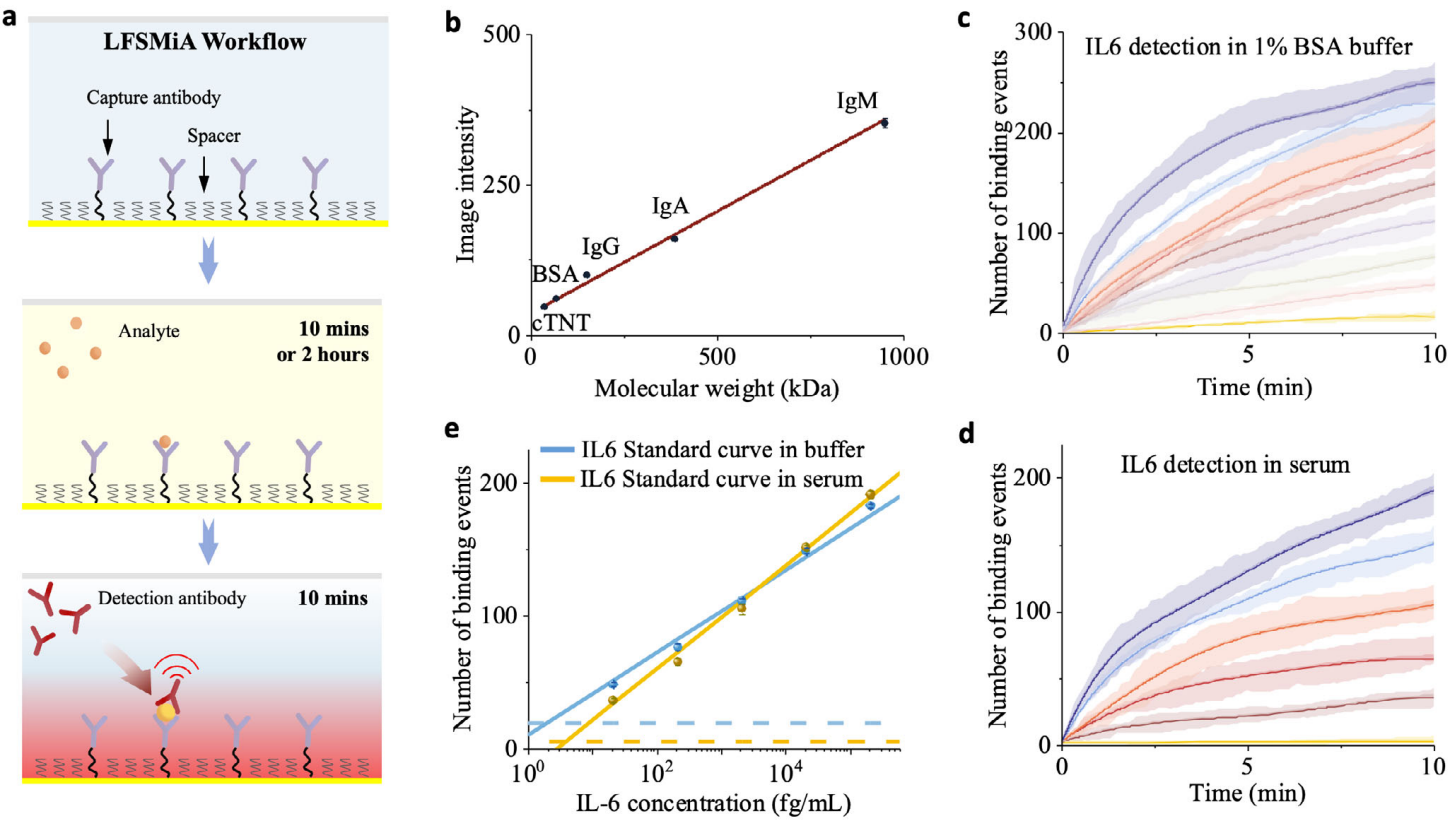
Validation of LFSMiA. a) Workflow of LFSMiA. Capture antibodies were first immobilized on the gold film through an alkane linker. Analyte samples flowed through the microfluidic channel for 10 min in NT‐proBNP detection or 2 h in the other cases. A 10 nM detection antibody solution was injected for 10 min, and the binding of the detection antibody to analytes captured on the sensor surface was recorded. b) The relationship between PSM image intensity and molecular weight (MW) of particles was determined from PSM images of different proteins in PBS solution. The mean intensity of one type of protein is the mean of its Gaussian fit to the corresponding histograms in Figure S2 (Supporting Information). The error bars are the model fitting error in Figure S2 (Supporting Information). The number of total binding events over time for different IL‐6 concentrations in c) pure buffer and (d) bovine serum. For clarity, the BGP model fitted mean values (solid lines) and the posterior standard deviations (dark‐colored shadows), and the sample standard deviations from the three replicates (light‐colored shadows) are plotted. Raw data of individual measurements can be found in Figures S4,S5 (Supporting Information). e) Standard curves of IL‐6 detection in pure buffer (blue) and bovine serum (yellow). The error bars are the posterior standard deviation calculated by the BGP model. The blue and yellow dashed lines represent the LOD for pure buffer and bovine serum, respectively, defined as mean plus three times the posterior standard deviation of the blank solution signal.

In LFSMiA, individual biomarkers of interest were pulled down by a capture antibody immobilized on a gold‐coated glass slide via a polyethylene glycol (PEG) linker (Figure 1a). The surface density between the PEG linker and spacer was modified to avoid the steric hindrance effect (Figure 2a). Detection antibodies were then introduced to bind with the biomarkers caught by the antibody, and the binding process was read out by PSM (Figure S1, Supporting Information).^[^ ^9^ ^]^ *p*‐polarized laser light was directed onto the gold surface via a prism to excite surface plasmon resonance (SPR) and the associated evanescent field. The scattered light within the field was collected by an objective placed on top of the microchip and imaged with a complementary metal oxide semiconductor (CMOS) camera, which recorded the dynamic binding process of individual detection antibody molecules with spatial resolution at the diffraction limit and a temporal resolution of ≈10 ms (Figure 1a,b).

A multi‐step image analysis approach was developed to track the dynamic information of the individual molecular binding events. Based on the position, duration, and intensity of the binding events, the specific events of the detection antibody binding to the capture antibody‐marker complex were identified and counted over time. As shown in Figure S11 (Supporting Information), first, the noise in the image was reduced by an n frame rolling averaging in the raw video starting at frame m, $N_{m}^{m+n}$, and then normalizing each frame of the averaged video in terms of their mean pixel value to avoid the intensity change from the light source (see Note S1, Supporting Information for more details). Next, a differential image sequence ($N_{m+n}^{m+2n}-N_{m}^{m+n}$) was obtained from the normalized image sequence by subtracting each frame from the following frame, which removed the static background and revealed binding or unbinding events. Second, a probability image (PI) sequence was obtained by convoluting the differential image sequence with Haar‐like array, which would reveal the morphological characteristics of the particle.^[^ ^10^ ^]^ The candidate pixels were selected if their intensity was higher than the mean intensity + 3 × standard deviation (s.d.) of the whole PI. Third, a fixed region was extracted in the differential images at positions of the candidate pixels mapped from the PI and fitted with a 2D Gaussian model to obtain the precise particle center location and intensity.^[^ ^11^ ^]^ The candidate pixels with a fitting bias too far from a Gaussian blob would be deleted.^[^ ^12^ ^]^ Last, as the binding of a particle appears as a brightening Gaussian blob and then gradually disappears (Figure S12, Supporting Information), the intensity of the single particle was determined as the peak of its temporal intensity profile. As shown in Figure 2b, the particle intensity in PSM images is linearly proportional to the molecular weight for different‐sized proteins due to the interference between the protein scattered light and the background scattered light.^[^ ^9^, ^13^ ^]^ Using this information, we determined whether a bound single particle was a detection antibody based on its image intensity. The binding events were considered nonspecific if they happened at the same location multiple times (Note S2, Supporting Information). The difference between specific and nonspecific binding is shown in the supporting videos. These analyses are critical for removing confounding effects caused by complex sample matrices like blood and greatly improve the counting accuracy for specific single molecule binding events.

A unique advantage of LFSMiA is the real‐time recorded binding events, which enable more precise measurement compared to endpoint counting methods. Viewing the specific binding event as a random event over time, stochastic process modeling is a natural choice to recover the random binding event at any time point.^[^ ^14^ ^]^ Considering that the binding counts change continuously and present dependence across time, we used a Bayesian Gaussian process model to incorporate the dependency between binding counts to improve the estimation accuracy.^[^ ^15^ ^]^ A Bayesian hierarchical model built on the Gaussian process was used to fit the real‐time binding counts (Note S3, Supporting Information), and the estimated values of the binding counts were provided along with their quantified posterior standard deviations. Finally, a standard curve was obtained by fitting the model estimated total binding events versus the spiked biomarker concentrations (Figure 1b). The model dramatically improved the assay precision and detection limit, and the details are presented in the discussion section below.

### 2.2 Validation of Label‐Free Assay by IL‐6 Detection

To assess the performance of LFSMiA, we first detected IL‐6, a key factor in hematopoiesis, immunomodulation, and inflammation processes,^[^ ^16^ ^]^ in pure buffer (1x PBS containing 1% BSA) and bovine serum. A series of standard buffer solutions or spiked bovine serum samples with different concentrations of IL‐6 flowed through the microfluidic channel for 2 h to react with the capture antibody pre‐functionalized on the sensor surface. Next, 10 nM detection antibody was injected for 10 min to bind to the IL‐6 captured by the surface antibody, and the process is monitored by PSM in real time (Figure 2a). The number of IL‐6 caught on the sensor surface was tracked by counting the binding events of the detection antibody with a temporal resolution of ≈10 ms. The temporal profile of the binding events follows the association kinetics of binding and correlates with the concentration of IL‐6 (Figure 2c,d).

The standard curves of IL‐6 detection in pure buffer and bovine serum were determined with 3 replicates for each concentration (Figure 2e). Defined as the estimated counts of blank experiments plus three times its corresponding s.d., the limit of detection (LOD) was calculated to be 1.89 and 3.91 fg mL^−1^ (0.09 and 0.19 fM) in pure buffer and bovine serum, respectively. The level of the nonspecific binding event in bovine serum was lower than in pure buffer, likely because bovine serum blocks the sensor surface better. For clarity, only part of the data was shown in Figure 2c‐e, and complete data is provided in the supporting information. We have realized an eight‐log detectable dynamic range, from 1.89 fg mL^−1^ to 0.21 µg mL^−1^ (Figure S3, Supporting Information). Two major advantages of LFSMiA contributed to the sub‐femtomolar sensitivity and wide dynamic range: i) most nonspecific binding events are removed based on the intensity, duration, and morphology of the binding events. ii) dynamic tracking of particle binding events is not limited by spatial resolution because it can temporally resolve binding signals when the surface is crowded.

### 2.3 Rapid NT‐proBNP Detection in Patient Serum Samples

**Figure 3:**
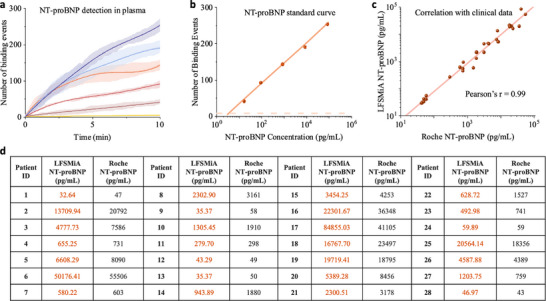
Rapid NT‐proBNP detection in human plasma and clinical evaluation. a) Temporal profiles for different NT‐proBNP concentrations in human plasma. For clarity, the BGP model fitted mean values (solid lines) and the posterior standard deviations (dark‐colored shadows), and the sample standard deviations from the three replicates (light‐colored shadows) are plotted. Raw data of individual measurements can be found in Figure S6 (Supporting Information). b) Standard curve of NT‐proBNP detection in human plasma. The error bars are the posterior standard deviation calculated by the BGP model. The dashed line represents the limit of detection for NT‐proBNP in human plasma, defined as mean plus three times the posterior standard deviations of blank solution (horse serum) signal. c) Pearson's correlation between LFSMiA and Roche's Elecsys proBNP II assay (r = 0.990). The results were determined from measurements of serum samples from 28 patients using both the Elecsys proBNP II assay and LFSMiA. d) Summary of all the measurement results for the 28 patients.

Simple, rapid, and sensitive assays are always needed in clinical settings.^[^ ^17^ ^]^ To show the applicability of LFSMiA for clinical use, it was evaluated for NT‐proBNP detection, a key clinical biomarker of heart failure.^[^ ^18^ ^]^ As NT‐proBNP naturally presents in human blood circulation, we first measured the baseline level of NT‐proBNP in the pooled human plasma, which was determined to be 8.23 pg mL^−1^. A series of standard samples with different concentrations of NT‐proBNP were prepared by spiking recombinant NT‐proBNP into the human plasma pool with the baseline level corrected. To evaluate the background level, horse serum was used as a blank control without human NT‐proBNP. Similar to IL‐6 experiment, these standard samples were introduced into the microfluidic channel for 10 min (Figure 2a). Then, 50 nM detection antibody was injected for 10 min. 50 nM was selected as it could amplify the sensor response but keep the nonspecific binding low enough (Figure S16, Supporting Information). The same PSM setup and parameters as IL‐6 detection were used. The temporal profiles of the binding event are shown in Figure 3a. After repeating the experiments 3 times, the standard curves of NT‐proBNP detection in human plasma were determined (Figure 3b). The LOD was determined to be 3.39 pg mL^−1^.

To evaluate the translational potential of LFSMiA for diagnostic applications, a cross‐validation between Roche's Elecsys proBNP II assay, an FDA‐approved routine clinical diagnostic testing for heart failure, was conducted. Serum samples from 28 patients were measured by both the Elecsys proBNP II assay and LFSMiA. The LFSMiA measured concentrations were highly correlated with those of the Elecsys proBNP II assay (Figure 3c, s1ope = 1.04 and R > 0.99), indicating a high potential of our method for point‐of‐care (POC) applications. More details can be found in Table S3 (Supporting Information). Overall, we have realized sub‐picomolar detection with a 20‐min total assay time. The dynamic range and detection limit of LFSMiA also satisfy the requirement for clinical NT‐proBNP testing.

### 2.4 Protein Biomarker Detection in Undiluted Whole‐Blood

As modern medicine greatly depends on clinical testing for disease diagnosis and monitoring, a simple assay for direct analysis of whole blood components is always preferred.^[^ ^19^ ^]^ Direct whole‐blood detection not only eliminates the loss of biomarkers during sample preparation but also minimizes total assay time. Current single‐molecule technologies, like SiMoAs and single‐molecule fluorescence microscopy,^[^ ^4^, ^5^ ^]^ still require the removal of blood cells or dilution of whole blood to achieve reliable detection due to matrix effects or extreme background autofluorescence. LFSMiA enables single‐molecule biomarker detection in undiluted whole blood due to four important features: 1) Microfluidic flow reduces the sensor blocking by blood cells. 2) Label‐free detection eliminates the photobleaching and background autofluorescence issues. 3) The two‐step assay separates signal readout from biomarker surface capture in whole blood, enabling reliable detection of single binding events. 4) Real‐time PSM imaging and dynamic tracking remove nonspecific background signals and reduce matrix effects.

**Figure 4:**
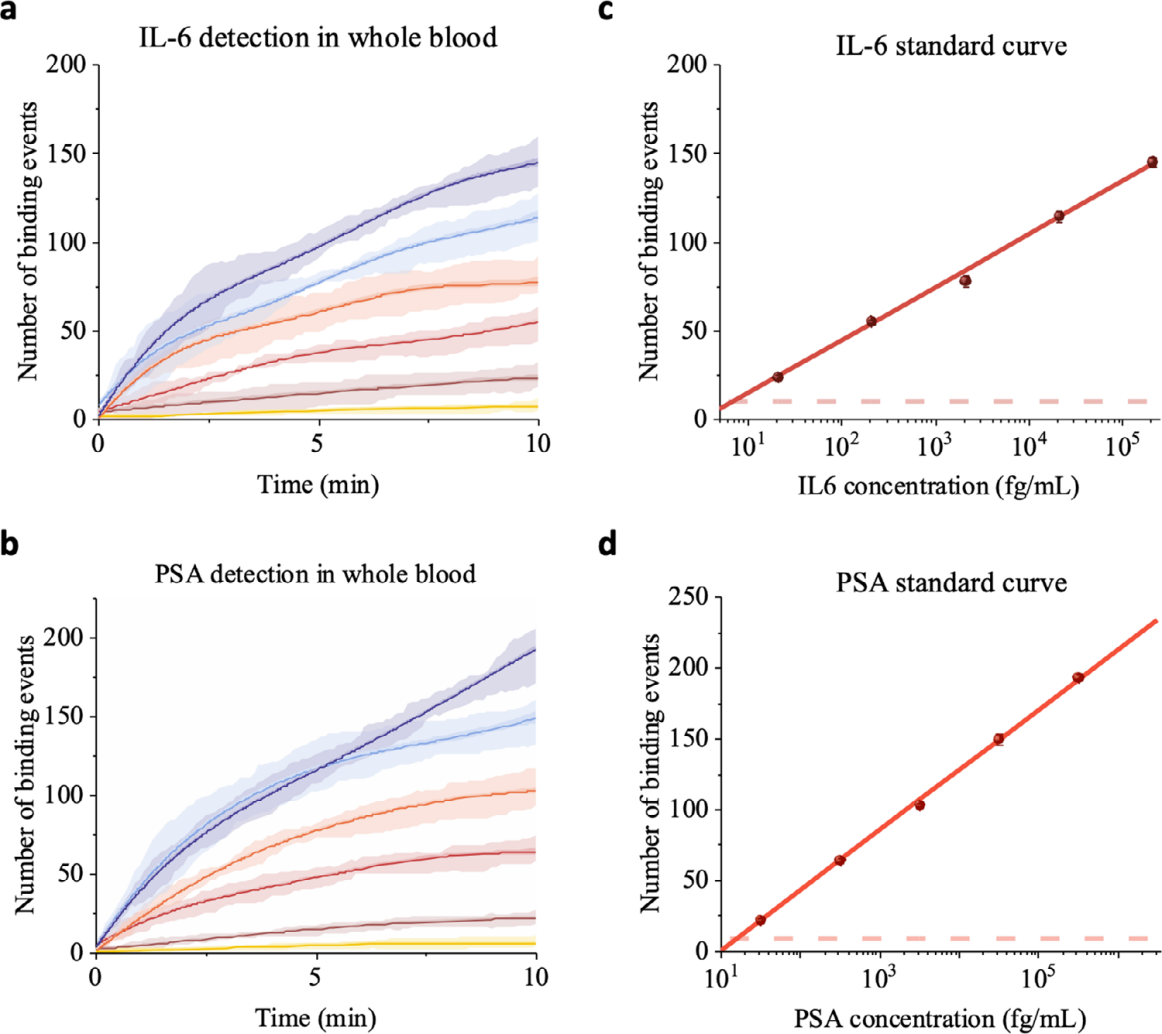
Protein detection in whole blood. Time courses of total binding events for different concentrations of a) IL‐6 and b) PSA in bovine whole blood. For clarity, the BGP model fitted mean values (solid lines) and the posterior standard deviations (dark‐colored shadows), and the sample standard deviations from the three replicates (light‐colored shadows) are plotted. Raw data of individual measurements in Figures S7,S8 (Supporting Information). Standard curve of c) IL‐6 and d) PSA in bovine whole blood. The error bars are the posterior standard deviations calculated by the BGP model. The red dashed line represents the limit of detection, defined as the mean plus three times the posterior standard deviations of the blank solution signal.

To validate the capability of LFSMiA for direct detection in whole blood, we measured IL‐6 and PSA in spiked whole blood samples.^[^ ^16^, ^20^ ^]^ Bovine whole blood was spiked with varying concentrations of IL‐6 and PSA and measured by LFSMiA, in the same two‐step assay for IL‐6 detection. The temporal profiles for IL‐6 and PSA detection are shown in Figure 4a,b, respectively. Standard curves for both biomarkers were generated after performing the experiments in triplicate (Figure 4c,d). The LOD for PSA in whole blood was determined to be 16.27 fg mL^−1^ (0.51 fM), while that for IL‐6 was 7.36 fg mL^−1^ (0.35 fM). The difference in slopes of the two standard curves should be due to the variance in affinity between the respective antibody pairs.

These results confirm the feasibility and robust performance of the LFSMiA for ultra‐sensitive biomarker detection in complex samples. To our knowledge, this is the first digital protein biomarker detection method using a label‐free single‐molecule technology. LFSMiA exhibits sensitivity comparable to other single‐molecule assays but eliminates the cost and issues of labeling. In addition, LFSMiA has the advantage of detecting individual biomarkers directly in undiluted complex clinical fluids, including serum, plasma, and whole blood—a capability not previously reported in single‐molecule technologies.

### 2.5 Point‐of‐Care LFSMiA

**Figure 5:**
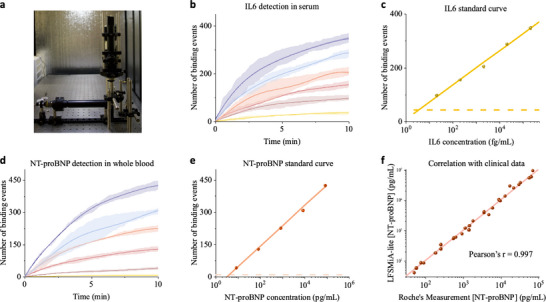
Point‐of‐Care LFSMiA and its clinical evaluation. a) Photograph of LFSMiA‐lite. b,d) are dynamic response profiles for different concentrations of IL‐6 in horse serum and NT‐proBNP in bovine whole blood, respectively. For clarity, the BGP model fitted mean values (solid lines) and the posterior standard deviations (dark‐colored shadows), and the sample standard deviations from the three replicates (light‐colored shadows) are plotted. Raw data of individual measurements can be found in Figures S21,S22 (Supporting Information). Stand curves of IL‐6 detection in horse serum c) and NT‐proBNP detection in bovine whole blood e). The error bars are the posterior standard deviations calculated by the BGP model. The dashed line represents the limit of detection, defined as the mean plus three times the posterior standard deviations of the blank solution signal. f) Pearson's correlation between NT‐proBNP concentration in patients' whole blood samples determined by LFSMiA‐lite and its corresponding serum samples measured using Roche's Elecsys proBNP II assay. Pearson's r = 0.997.

To assess the applicability of the LFSMiA in POC settings, we developed an affordable label‐free single‐molecule microscope (LFSMiA‐lite) with compact dimensions of 40 × 15 × 40 cm, constructed using commercially available optical components. The total cost of the setup is approximately USD 2000 (Figure 5a). Furthermore, we expanded the imaging area to achieve higher particle counts within 10 min, thereby enhancing the precision of the assay. To our knowledge, no commercially available microscopy system combines low cost, small size, and single‐molecule sensitivity in a label‐free format.

First, we established the relationship between molecular weight and particle image intensity using the new LFSMiA‐lite system, enabling the determination of threshold values for optimized data analysis (Figure S20, Supporting Information). To compare the performance of LFSMiA‐lite with the laboratory‐grade version, we selected IL‐6 as a model analyte. Following the same experimental protocol as the laboratory setup, LFSMiA‐lite demonstrated a similar LOD of 3.37 fg mL^−1^ (Figure 5c). These results suggest that the sensitivity and quantitative detection capability of LFSMiA‐lite remain uncompromised despite its smaller size and lower cost.

To demonstrate the clinical translational potential and POC applicability, we employed LFSMiA‐lite for rapid NT‐proBNP detection in whole blood. Bovine whole blood samples spiked with varying concentrations of NT‐proBNP were measured in triplicate using LFSMiA‐lite, following the same procedure used in previous NT‐proBNP detection experiments. The resulting temporal profiles are shown in Figure 5d. A standard curve was generated from these data, and the LOD was determined to be 4.14 pg mL^−1^ (Figure 5e). Additionally, whole blood samples from 28 patients were measured using LFSMiA‐lite, while the corresponding serum samples were analyzed with Roche's Elecsys proBNP II assay for cross‐validation. The results obtained with LFSMiA‐lite exhibited excellent correlation with the FDA‐approved assay, indicating the strong potential of this method for blood biomarker diagnostics in POC settings (Figure 5f).

## 3 Discussion

### 3.1 Using Real‐Time Data to Improve Detection Precision

Most digital immunoassay techniques focus on endpoint detection, relying on counting the total single‐molecule events at the end of the incubation/binding period. The inherent relative counting error is fundamentally limited by $\sqrt{N}$ (where N represents the total counts), which defines the digital noise constraint in such assays. One of the most significant distinctions between LFSMiA and existing assays is that LFSMiA operates as a real‐time assay. By fully utilizing both kinetic and statistical information with the BGP model, LFSMiA effectively surpasses the digital noise limitation inherent in traditional digital immunoassays by utilizing the time dependency of binding data to improve the detection precision and limit (Note S3, Supporting Information).

**Figure 6:**
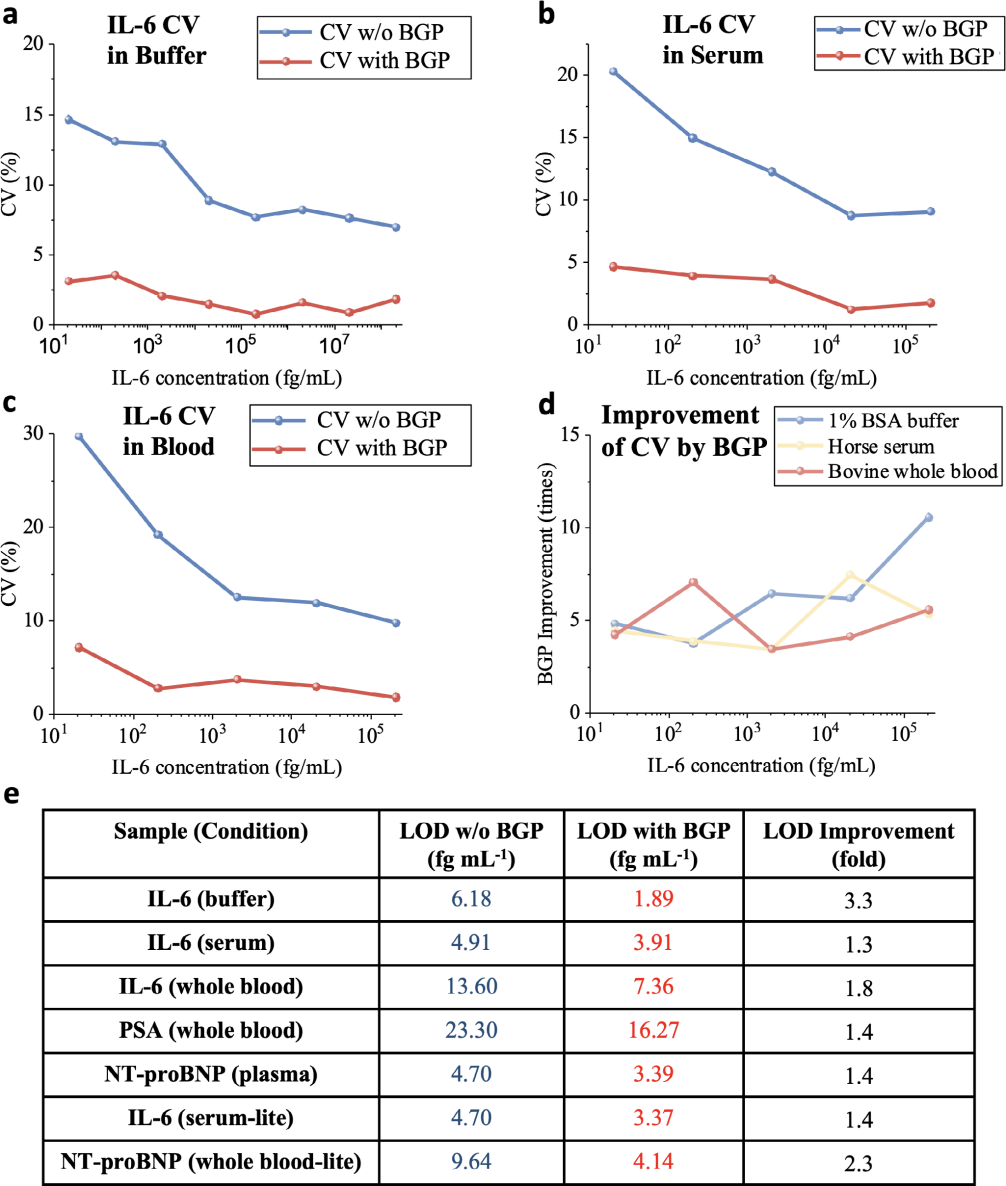
Improvement of detection limit and precision by the BGP model. a‐c) show the coefficient of variation of IL‐6 detection in pure buffer, horse serum, and bovine whole blood, determined with and without the model, respectively. d) presents the improvement of CV under different conditions by the BGP model. e) Comparison of the LOD obtained by the standard method and the BGP model under different samples and conditions.

To validate the assay performance improvements brought by the model, we evaluated the model's impact on the coefficient of variation (CV) and LOD. We first compared the CV obtained by the model and by the standard deviation of 3 replicates. Improvements in the CV for IL‐6 detection in pure buffer (1% BSA solution), horse serum, and whole blood are shown in Figure 6a‐c, respectively. The magnitude of improvement was calculated and presented in Figure 6e. Notably, we observed greater improvement at lower concentrations, where the total count is relatively low. Based on the results, the model yields an average CV improvement of over five‐fold. Since the LOD is related to the CV of blank signal (LOD is defined as mean + 3 × s.d. of blank signal), the LODs across all the samples we measured are also significantly lowered by the model, as shown in Figure 6e.

### 3.2 Additional Advantages of Real‐Time Counting

The real‐time counting method used in LFSMiA could overcome the counting limitation of endpoint assays for low‐concentration samples. LFSMiA reports the total binding events N(T) of the detection antibody on the surface as the output signal, which is defined as

(1) $$N\left(T\right)=\int_{0}^{T}K_{\mathrm{on}}{\left[A\right]}_{t}{\left[P\right]}_{t}\mathrm{dt}$$

where k_on_ is the association rate constant of the detection antibody, [A]_t_ and [P]_t_ are the concentrations of the free analyte and the detection antibody at time t. Therefore, the signal of LFSMiA would still increase even after detection antibody binding reaches dynamic equilibrium, which makes it superior to conventional endpoint assays for detecting low‐abundance biomarkers.^[^ ^21^ ^]^

### 3.3 Competitive Advantages

LFSMiA shows obvious advantages compared with existing label‐based digital immunoassays. The label‐free nature of LFSMiA avoids the cost associated with fluorescence probes. LFSMiA could be easily adopted on commercial SPR systems by adding an air objective to the top of the imaging area. The ability to detect individual biomarkers in undiluted complex clinical fluids simplifies the workflow of clinical testing and is POC compatible. Compared with label‐free methods like interferometric scattering mass spectrometry and SPR,^[^ ^22^ ^]^ LFSMiA excels at distinguishing the specific binding of detection antibody to the analyte versus nonspecifically absorbed biomolecules from the complex media. Therefore, we believe LFSMiA is a promising new tool that will have broad applications in biomedical research and medical diagnostics.

### 3.4 Limitations and Mitigations

The accuracy and sensitivity of LFSMiA are primarily limited by the number of binding events enumerated, which could be improved by increasing the imaging field‐of‐view (FOV) or reaction time. We compared the results for the reaction time of 5 min or 10 min, and the longer reaction time improves the accuracy and sensitivity (Figure S18, Supporting Information). While the clinically acceptable assay time and the effects of analyte dissociation restrict the length of reaction time, we could apply real‐time adaptive counting by stopping counting when a confidence threshold is reached to improve assay time. Increasing FOV requires more expensive optics and cameras with more pixels and higher data recording rates. A higher power light source is also needed for single‐molecule sensitivity in a larger FOV.^[^ ^23^ ^]^ Alternatively, image processing technologies powered by machine learning could detect IgG antibodies at a lower signal‐to‐noise ratio, enabling larger FOV imaging without a significant increase in hardware cost.^[^ ^24^ ^]^

In the current set‐up, LFSMiA uses an incident illumination intensity of ≈2 kW cm^−2^ to image a single detection antibody with an acceptable signal‐to‐noise ratio. A higher signal‐to‐noise ratio can improve the accuracy of dynamic counting of single molecule binding events, which could be obtained by increasing the incident light intensity. However, high illumination intensity could damage the protein molecules on the surface by the photothermal effect.^[^ ^25^ ^]^ Using a low light absorption glass slide coupled with evanescent scattering imaging could overcome this limit.^[^ ^26^ ^]^

As a sandwich immunoassay, the affinity and specificity of the antibodies will influence the performance of LFSMiA. Although pricy, most commercial monoclonal antibodies with nanomolar or lower K_D_ meet the specificity and affinity requirement of LFSMiA.^[^ ^27^ ^]^ Alternative affinity probes, such as aptamers and multivalent nanobodies,^[^ ^28^ ^]^ could overcome the limitations of monoclonal antibodies and further improve the affordability and performance of LFSMiA.

## 4 Conclusion

In this work, we have developed a label‐free digital kinetic immunoassay that addresses the limitations of current single‐molecule immunoassays, enabling specific, rapid, and ultrasensitive detection of molecular biomarkers in complex sample matrices, including undiluted serum, plasma, and whole blood. By integrating a BGP model, the assay fully exploits kinetic information to enhance sensitivity and precision. We validated the LFSMiA using a commercially available and well‐characterized IL‐6 antibody pair, achieving sub‐femtomolar detection in pure buffer, undiluted serum, and whole blood. The assay demonstrated an impressive dynamic range of eight logs, surpassing the performance of most existing technologies. The results of IL‐6 detection across different sample matrices reveal that interfering substances in real biological samples can impact the LOD and sensitivity of the assays. In addition, the LFSMiA for rapid NT‐proBNP detection in plasma exhibited superior sensitivity, a broader dynamic range, and a significantly shorter sample‐to‐result time than the gold‐standard ELISA. When applied to serum samples from heart failure patients, the LFSMiA showed excellent correlation with FDA‐approved routine clinical diagnostic tests, demonstrating significant potential for clinical translation. The successful ultra‐sensitive detection of multiple clinically important biomarkers in undiluted whole blood highlights the potential of the LFSMiA for POC testing, as it eliminates the need for sample preparation and the associated risk of biomarker loss. The inexpensive, compact label‐free microscope (LFSMiA‐lite) we developed for digital immunoassays has proved to be as effective as the laboratory‐based microscopy. Applied to whole blood samples from heart failure patients, LFSMiA‐lite, using a 40 µL sample volume and a total assay time of 20 min, produced results that strongly correlated with those obtained using clinical diagnostic assays. Thus, the LFSMiA is a promising platform for biomedical research and clinical diagnostics and offers significant potential for POC diagnostics.

## 5 Experimental Section

### Reagents and Materials

The recombinant antigen (catalog No. 8RTT5) for human cardiac troponin T was purchased from HyTest Ltd. Bovine serum albumin (BSA, catalog no. A7638‐5G) and immunoglobulin G (IgG, catalog no. I2511‐10MG) were purchased from Sigma‐Aldrich. Human colostrum immunoglobulin A (IgA, catalog no. SIA1901‐R22) and human plasma immunoglobulin M (IgM, catalog no. IP2020‐03) were purchased from Athens Research and Technology. IL‐6 monoclonal capture antibody (clone no. MQ2‐13A5, catalog no. 14‐7069) and detection antibody (clone no. MQ2‐39C3, catalog no. 13‐7068) were purchased from ThermoFisher Scientific. Recombinant human IL‐6 protein (catalog no. 206‐IL) was purchased from R&D Systems. PSA monoclonal capture antibody (clone no. M612165, catalog no. 10‐P21A), detection antibody (clone no. M612166, catalog no. 10‐P20A) and purified native human PSA protein (catalog no. 30‐1205) were purchased from Fitzgerald Industries. Human NT‐proBNP capture antibody (catalog no. BRJNBNPS108), detection antibody (catalog no. BRJNBNPS102) and recombinant protein (catalog no. GRCBNPS101) were purchased from Fapon International Limited. Reagent diluent (catalog no. DY995), streptavidin conjugated horseradish peroxidase (streptavidin‐HRP, catalog no. DY998), stop solution (catalog no. DY994) and high‐binding microplate (catalog no. DY990) were purchased from R&D Systems. Gender unspecified pooled K2EDTA plasma from healthy human donors and gender unspecified pooled bovine whole blood were purchased from BioIVT Elevating Science. Heat‐inactivated horse serum (catalog no. 26050) was purchased from ThermoFisher Scientific. ACS grade denatured ethanol (catalog no. BDH1158) was purchased from VWR International. N‐hydroxysuccinimide (NHS, catalog no. 130672), N‐(3‐dimethylaminopropyl)‐N‐ethylcarbodiimide hydrochloride (EDC, catalog no. 03450), Amicon Ultra‐0.5 mL centrifugal filters, 6‐mercapto‐1‐hexanol (catalog no. 725226), 3‐(N‐Morpholino)propanesulfonic acid (MOPS), phosphate buffered saline (PBS), and 8‐mercaptooctanoic acid (catalog no. 675075) were purchased from Sigma‐Aldrich (St. Louis, MO). Sylgard 184 Clear Silicone Elastomer Kit (catalog no. DC4019862) was purchased from Krayden Inc.

### Microfluidic Sensor Chip Fabrication

47 nm gold‐coated 24 × 50 mm^2^ glass coverslips were used as the substrates of the sensor chips. The gold‐coated substrate was rinsed with ethanol and deionized water sequentially three times and annealed using hydrogen flaming. The annealed gold substrate was immersed overnight in an ethanol solution containing 100 µM 8‐mercaptooctanoic acid (MOA) and 10 mM 6‐mercapto‐1‐hexanol (MCH). The coated substrate was thoroughly rinsed with ethanol and deionized water, and then a layer of 50 µm thick double‐sided tape with a 3 × 36 mm^2^ straight channel was adhered to the gold surface. Then, a 24 × 40 mm^2^ glass coverslip with two drilled holes located at the two ends of the straight channel was placed onto the double‐sided tape and pressed to form an enclosed microfluidic channel. To enable fluid dispensing, two polydimethylsiloxane (PDMS) pieces with a punched hole were bonded to the top surface of the glass coverslip and aligned the holes with the holes on the glass to form an inlet and an outlet. Finally, epoxy glue was applied to all the edges of the top glass coverslip and the 2 PDMS pieces and cured for 1 h at room temperature to secure and seal the sensor chip.

### Sensor Surface Functionalization

The capture antibodies for IL‐6, NT‐proBNP, and PSA were preprocessed by buffer exchanging into MOPS buffer (5 mM MOPS, pH 7.4) with 5 spin cycles using Amicon centrifugal filters. 100 µL of an aqueous solution containing 5 mM EDC and 10 mM NHS was injected into the microfluidic channel and incubated for 5 min. Later, this procedure was repeated two more times to activate the carboxylic acid functional groups on the sensor surface. The channel was flushed with 100 µL MOPS buffer. Next, in 1 h total incubation time, 20 µL of 100 µg mL^−1^ capture antibody in MOPS buffer was injected three times at 0, 5, and 15 min. Then, 100 µL aqueous solution containing 1 M ethanolamine with pH of 9.6 was injected to quench the unreacted NHS esters. Lastly, 100 µL of 1x PBS was injected to flush the channel. The sensor chips were prepared prior to experiments and used within the same day.

### Human IL‐6 and PSA Detection

For IL‐6 detection in pure buffer, human recombinant IL‐6 was spiked into 1x reagent diluent (1% BSA in 1x PBS) to reach final concentrations of 1, 10, 100, 10^3^, 10^4^, 10^5^, 10^6^, and 10^7^ fM. For IL‐6 detection in horse serum, 1, 10, 100, 10^3^, and 10^4^ fM of recombinant IL‐6 were spiked into the horse serum. For IL‐6 or PSA detection in bovine whole blood, the recombinant proteins of IL‐6 or PSA were spiked to final plasma concentrations of 1, 10, 100, 10^3^, and 10^4^ fM. The corresponding sample matrices without spiking were used as blank controls to measure their background level. These spiked samples were injected through the sensor chips for 2 h with a 10 µL/min flow rate. After being flushed with 1x PBS, the chips were installed onto PSM. 3 mL of 10 nM detection antibody in 1x PBS flowed through the microfluidic channel for 10 min, driven by a gravity pump, while PSM recorded images of the sensor surface in real‐time. Finally, an in‐house dynamic tracking algorithm analyzed the PSM images to count the detection antibodies binding to the sensor surface.

### Human NT‐proBNP Detection and Clinical Evaluation

To generate the NT‐proBNP calibration curve in human plasma and bovine whole blood, recombinant human NT‐proBNP protein was spiked into the pooled human plasma or bovine whole blood to concentrations of 1, 10, 100, 10^3^, 10^4^, and 10^5^ pM. The endogenous level of NT‐proBNP in the human plasma pool was 0.97 pM, measured by conventional ELISA. As such, the corrected concentrations were 1.97, 10.97, 100.97, 10^3^, 10^4,^ and 10^5^ pM. These standard samples were injected through the sensor chips for 10 min with a flow rate of 50 µL/min and 5 µL/min for plasma and whole blood samples, respectively, and then measured by PSM following the same procedure as in IL‐6 detection. Under a protocol approved by the Mayo Clinic Institutional Review Board and Biospecimens Subcommittee (IRB # 19–002558/Bio00017399), 28 serum samples and 28 whole blood samples from different patients were deidentified and provided by Mayo Clinic Arizona and measured by the LFSMiA and LFSMiA‐lite, respectively. The patient samples were residual volume from routine clinical testing of NT‐proBNP by Roche's Elecsys proBNP II assay. They were transported in an icebox to our lab, stored at 4 °C, and tested within 6 days. The corresponding Roche assay results were used to validate LFSMiA and LFSMiA‐lite results.

### Conventional ELISA Measurement of NT‐proBNP Baseline

To measure the baseline NT‐proBNP level in the spiking human plasma pool, a 96‐well microplate was coated overnight with 100 µL of 2 µg mL^−1^ capture antibody of NT‐proBNP in 1x PBS. After being washed three times with 200 µL of 0.05% PBST (1x PBS containing 0.05% v/v Tween 20), the plate wells were blocked with 200 µL 1X reagent diluent for 1 h. Then, a series of horse serum standard samples with spike‐in NT‐proBNP concentrations of 25, 50, 100, and 200 pg mL^−1^ were prepared. Horse serum without spiking was used as the 0 pg mL^−1^ blank control. A mixture of 50 µL NT‐proBNP‐spiked horse serum standard sample or the human plasma and 50 µL biotinylated detection antibody (2 µg mL^−1^) was incubated in the wells for 1 h. Following another washing step as previously, 100 µL streptavidin‐HRP solution was incubated in the wells for 20 min. Washed again with 0.05% PBST, the wells were reacted with 100 µL TMB substrate solution for 20 min. Lastly, 50 µL stop solution was added, followed by reading the absorbance of each well using a microplate reader (EnVision 2104, Perkin Elmer).

### Experimental Setup

For the lab‐use PSM setup, a 660 nm,120 mW diode laser (L660P120, Thorlabs) was used as the light source. The light was collimated by a 20 × objective, with the beam size reduced by a lens group. The sized beam was then focused on the prism surface by a short‐focus lens with an incident angle of 71° to reach SPR. The scattered light from the biomolecule and gold surface was collected by a 60 × air objective (Olympus, LUCPLFLN60X, NA = 0.7) equipped with a 180 mm tube lens to form an image on a CMOS camera (MQ013MG‐ON, XIMEA). More details can be found in Figure S1 (Supporting Information).

For LFSMiA‐lite, the basic principle was similar to the lab‐use setup. However, we changed the light source to a low‐cost 100 mW diode laser ($70, 660nm‐RDM, Berlin Lasers, Hong Kong) and a 60 × air objective ($391, Olympus Ach 60x / 0.80, Infinity / 0.17 Microscope Objective). We also simplified the optics and the structure of the setup to minimize the size and cost of the LFSMiA‐lite setup.

### Image Processing

The raw image sequence was recorded by XIMEA CamTool and processed by custom‐written Matlab scripts and R codes (See Notes S1–S3, Supporting Information for additional details).

### Statistical Analysis

A 2D Gaussian model was used to fit the antibody binding spot extracted from the plasmonic images by custom‐written Matlab scripts, as detailed in the Principle of LFSMiA section and Note S1 (Supporting Information). A Bayesian Gaussian process model was used to analyze the real‐time binding events detected from the plasmonic scattering images by custom‐written R codes, see Note S3 (Supporting Information) for details. Pearson's correlation (OriginPro) was used to compare the results obtained by LFSMiA and Roche's Elecsys proBNP II assay for the patient samples.

## Conflict of Interest

The authors declare the following competing financial interest(s): S.W. is a member of the technology advisory board of Biosensing Instrument Inc. A US patent application (18/956,466) has been filed by the Arizona Board of Regents on behalf of Arizona State University based on an early draft of this article. The inventors are S.W., X.Z, and S.Z.

## Author Contributions

S.W. and X.Z. conceived the project. X.Z., G.M., and S.W. built the instrument. X.Z., C.C., and S.W. designed the experiments. X.Z. and C.C. carried out the experiments. X.Z., S.Z., J.J., X.Z., and S.W. analyzed the data. C.C., M.J.C., B.B., and Z.W. fabricated the gold coated sensor chips. C.S. and E.Y. provided the clinical samples and corresponding validation data. S.W. supervised the project. X.Z., C.C., S.Z., and S.W. wrote the paper, all authors reviewed the paper.

## Supporting information



Supporting Information

Supplemental Video 1

Supplemental Video 2

## Data Availability

The data that support the findings of this study are available from the corresponding author upon reasonable request.
