# Supplementary material for: Label‐Free Single‐Molecule Immunoassay
Source: Adv Sci (Weinh). 2025 Jun 20;12(31):e05207. doi: 10.1002/advs.202505207 (PMC12376538; doi:10.1002/advs.202505207)
Supplement: Supplementary file 1 — Supporting Information [file ADVS-12-e05207-s003.pdf]

ADVANCED SCIENCE
Open Access

Supporting Information

for *Adv. Sci.*, DOI 10.1002/advs.202505207

Label-Free Single-Molecule Immunoassay

*Xiaoyan Zhou, Chao Chen, Shuang Zhou, Guangzhong Ma, Mohammad Javad H. N. Chemerkouh, Christine L. H. Snozek, Eric H. Yang, Jiapei Jiang, Brandyn Braswell, Zijian Wan, Xinyu Zhou and Shaopeng Wang**

Supporting Information

Label-free Single-molecule Immunoassay

Xiaoyan Zhou[1,2], Chao Chen[1,3], Shuang Zhou[4], Guangzhong Ma[1], Mohammad Javad H. N. Chemerkouh[1,2], Christine LH Snozek[5], Eric H. Yang[6], Jiapei Jiang[1], Brandyn Braswell[1,7], Zijian Wan[1,2], Xinyu Zhou[1,3], Shaopeng Wang[1,3]*

[1]Center for Bioelectronics and Biosensors, The Biodesign Institute, Arizona State University, Tempe, Arizona 85287, United State
[2]School of Electrical, Computer and Energy Engineering, Arizona State University, Tempe, Arizona 85287, United State
[3]School of Biological and Health Systems Engineering, Arizona State University, Tempe, Arizona 85287, USA.
[4]School of Mathematical and Statistical Sciences, Arizona State University, Tempe, Arizona 85287, United State
[5]Department of Laboratory Medicine and Pathology, Mayo Clinic Arizona, Phoenix, Arizona 85054, United State
[6]Department of Cardiovascular Disease, Mayo Clinic Arizona, Phoenix, Arizona 85054, United State
[7]School of Engineering for Matter, Transport and Energy, Arizona State University, Tempe, Arizona 85287, United State

*Corresponding author email: Shaopeng.Wang@asu.edu

Figures S1 – S26

Tables S1 – S3

Notes S1 – S7

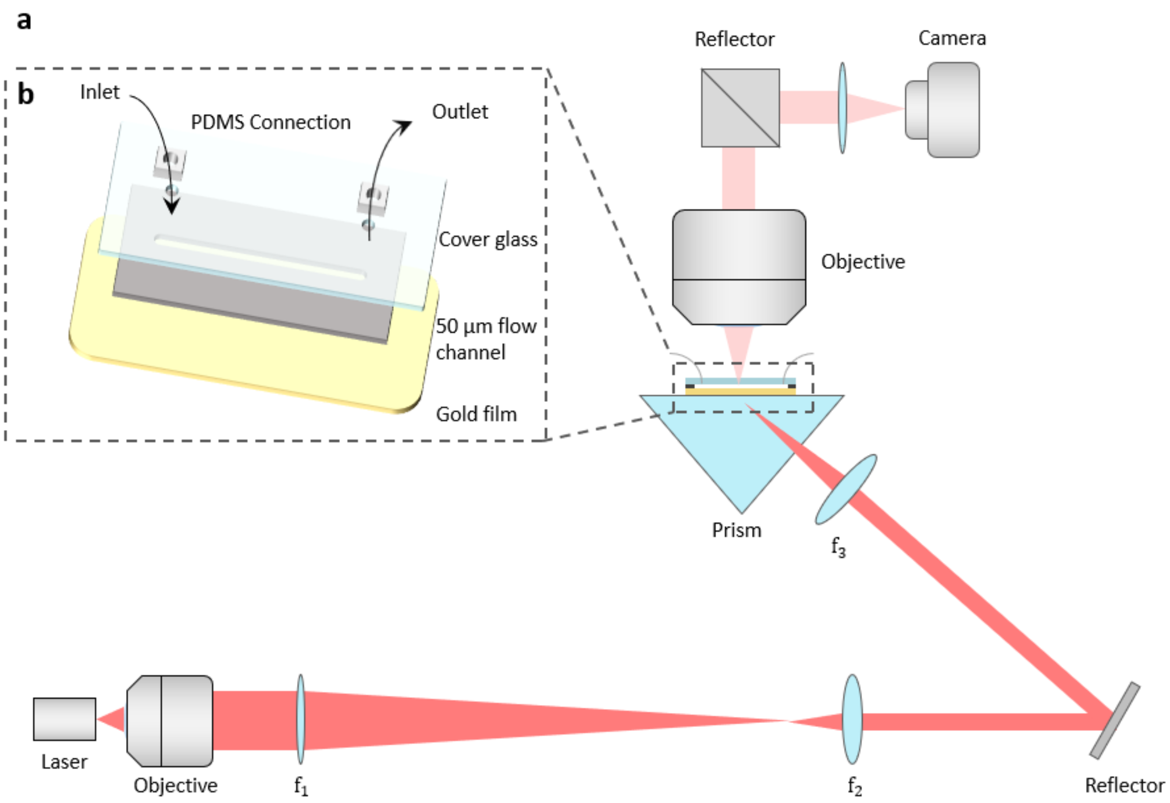

Figure S1. The setup for LFSM-immunoassay. (a) Optical configuration of plasmonic scattering microscopy used for LFSM-immunoassay. The laser light (660 nm diode laser, up to 120 mW, L660P120, Thorlabs) is collected and collimated by a 20× objective. The beam size is reduced by a lens group (f1 and f2) to increase light intensity. The incident light is directed and focused on the prism surface with an incident angle of 71° to excite SPR by a reflector and a short-focus lens (f3). The focal lengths for the lenses are $f_1 = 200\ \mathrm{mm}$, $f_2 = 30\ \mathrm{mm}$, and $f_3 = 30\ \mathrm{mm}$. The intensity of incident light is up to 2000 W/cm^2. The scattered light from the biomolecule and gold surface is collected by a 60× air objective (Olympus, LUCPLFLN60X, NA = 0.7) equipped with a 180 mm tube lens to form an image on a CMOS camera (MQ013MG-ON, XIMEA). (b) The microfluidic channel assembly. A 50 µm thick double-sided tape with a 3×36 mm^2 straight channel is sandwiched between a cover glass and a gold film. The gold film is pre-modified with alkane linker and spacer. The cover glass has two drilled holes located at the two ends of the straight channel, and two PDMS pieces each with a through hole are adhered to the cover glass to form the inlet and outlet of the microfluidic channel. The microfluidic channel assembly is sealed with epoxy glue.

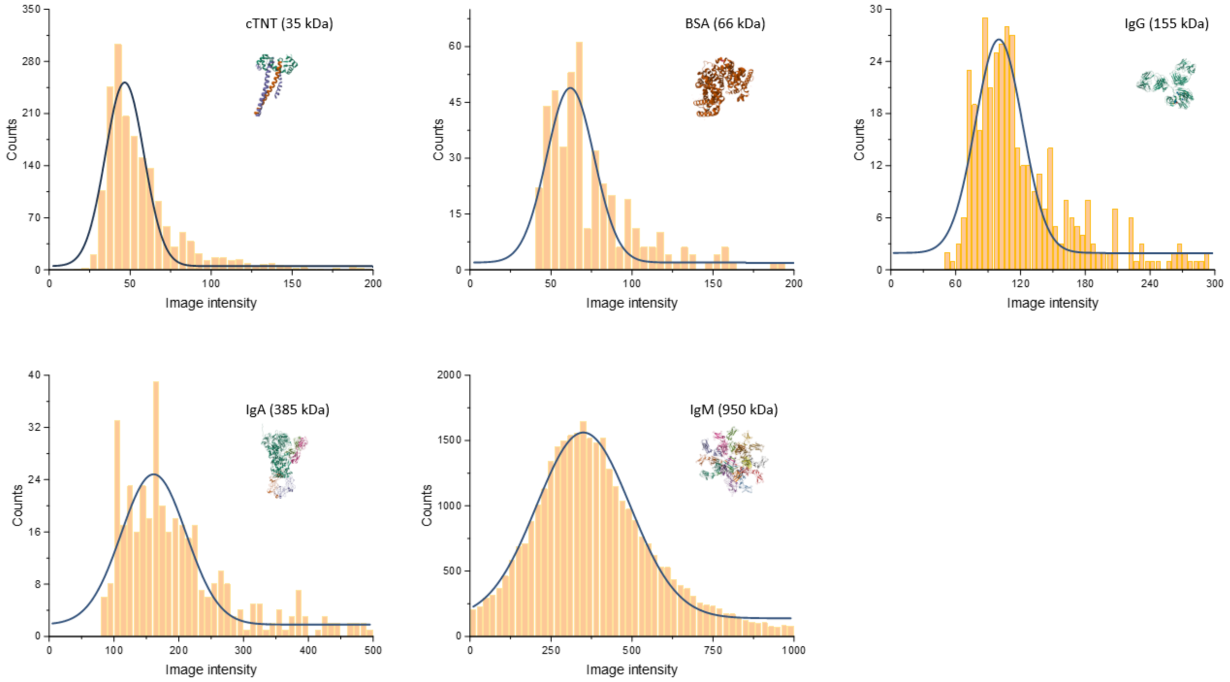

Figure S2. Calibration data of PSM mass detection. To determine the relationship between PSM image intensity and protein molecular weight, 5 different proteins with known molecular weight were dissolved in PBS buffer and flowed over the bare gold surface. Binding events of different proteins were recorded and extracted to obtain histograms of their image intensity. The solid lines are the Gaussian fitting results for the 5 proteins. The incident light intensity was 2 kW/cm^2. The exposure time was 1 ms for IgM, 2 ms for IgA, 5 ms for IgG, 10 ms for BSA, and 15 ms for cTnT. All the results were normalized to an exposure time of 10 ms.

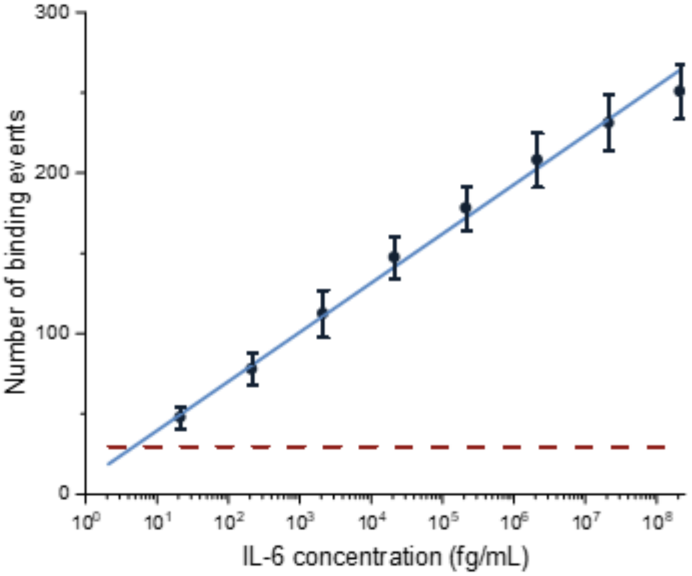

Figure S3. Complete standard curve of IL-6 detection in pure buffer, where the error bars were calculated from s.d. of triplicate tests. The dashed line is the limit of detection for IL-6 detection in pure buffer, defined as mean + 3×s.d. counts of blank solution.

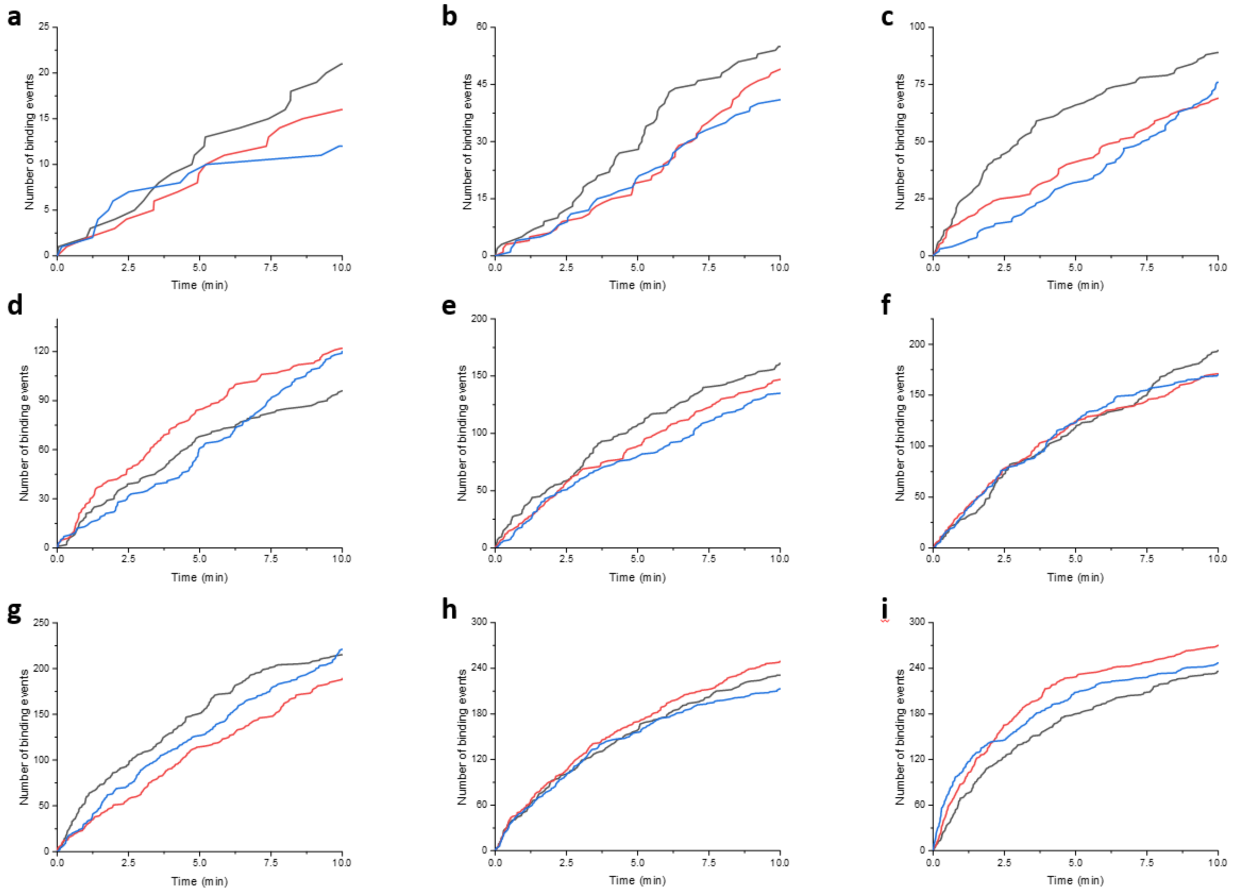

Figure S4. Temporal profiles of total binding events for the 3 replicate tests of different IL-6 concentrations in the pure buffer. (a–i) The real-time counting results of total binding events for IL-6 spiked pure buffer with concentrations of 0, 21 fg/mL, 210 fg/mL, 2.1 pg/ml, 21 pg/mL, 210 pg/mL, 2.1 ng/mL, 21 ng/mL and 210 ng/mL respectively.

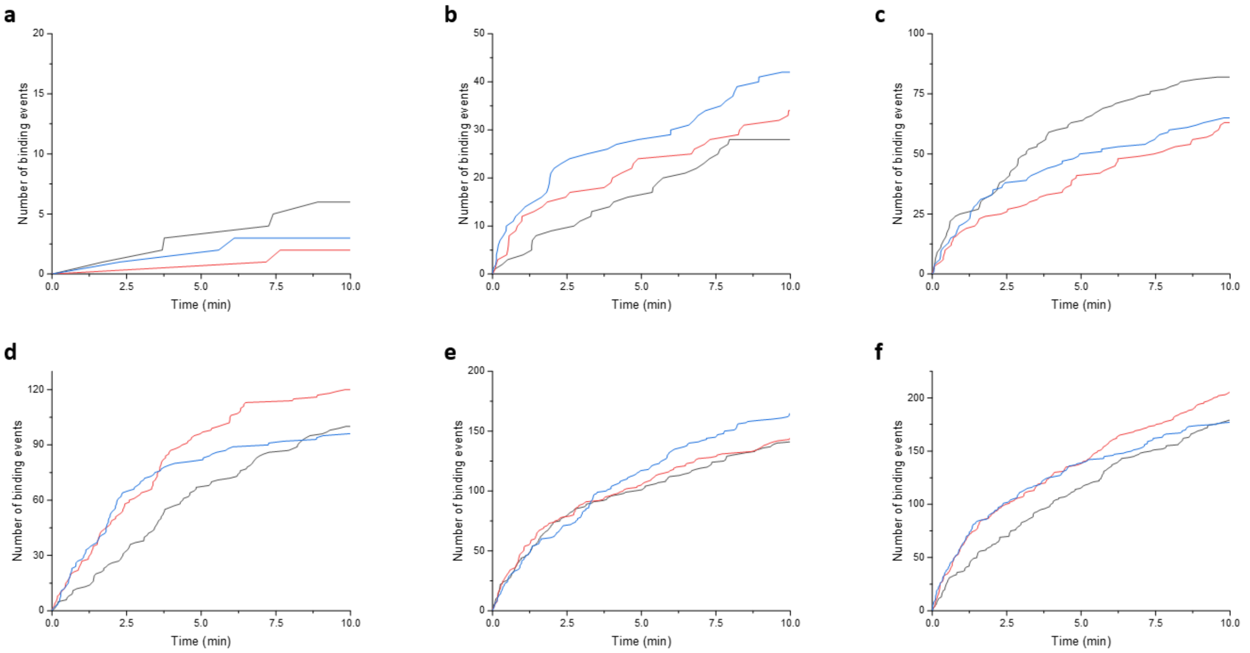

Figure S5. Time courses of total binding events for triplicate tests of different IL-6 concentrations in bovine serum. (a–f) The real-time counting results of total binding events for IL-6 spiked bovine serum at 0, 21 fg/mL, 210 fg/mL, 2.1 pg/ml, 21 pg/mL, and 210 pg/mL, respectively.

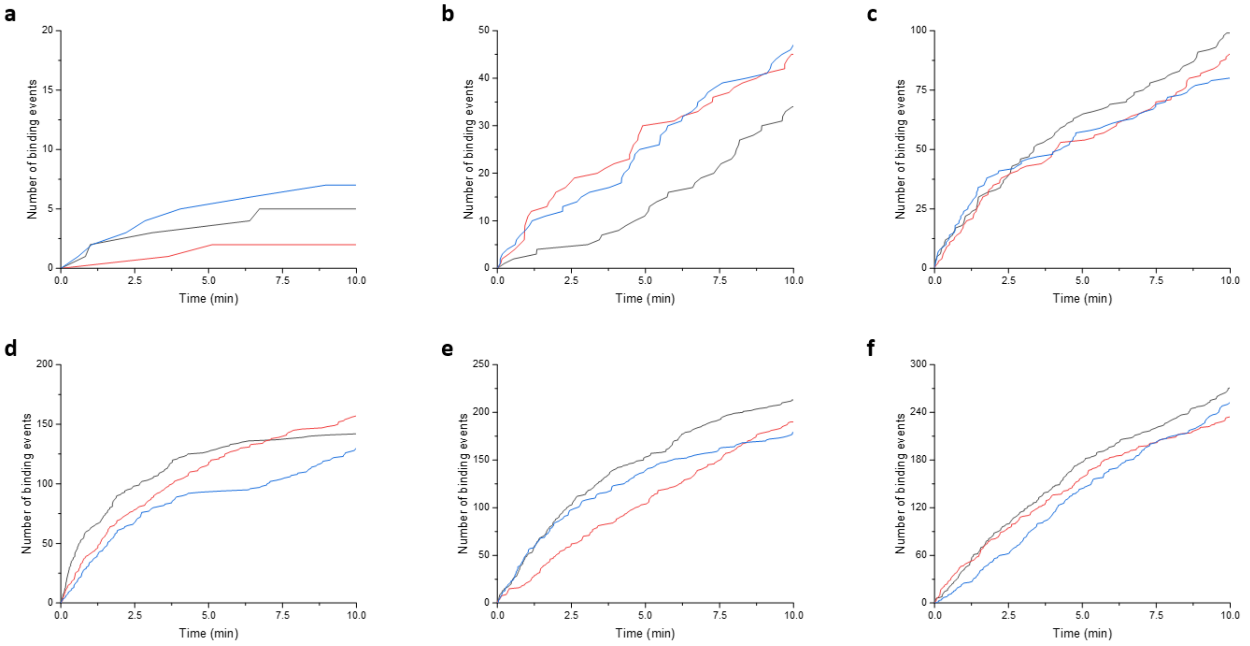

Figure S6. Real-time counting results of total binding events for generating NT-proBNP standard curve corresponding to (a) blank buffer (horse serum), NT-proBNP spiked human plasma with concentrations of (b) 16.7 pg/mL, (c) 93.2 pg/mL, (d) 850 pg/ml, (e) 8.5 ng/mL and (f) 85 ng/mL.

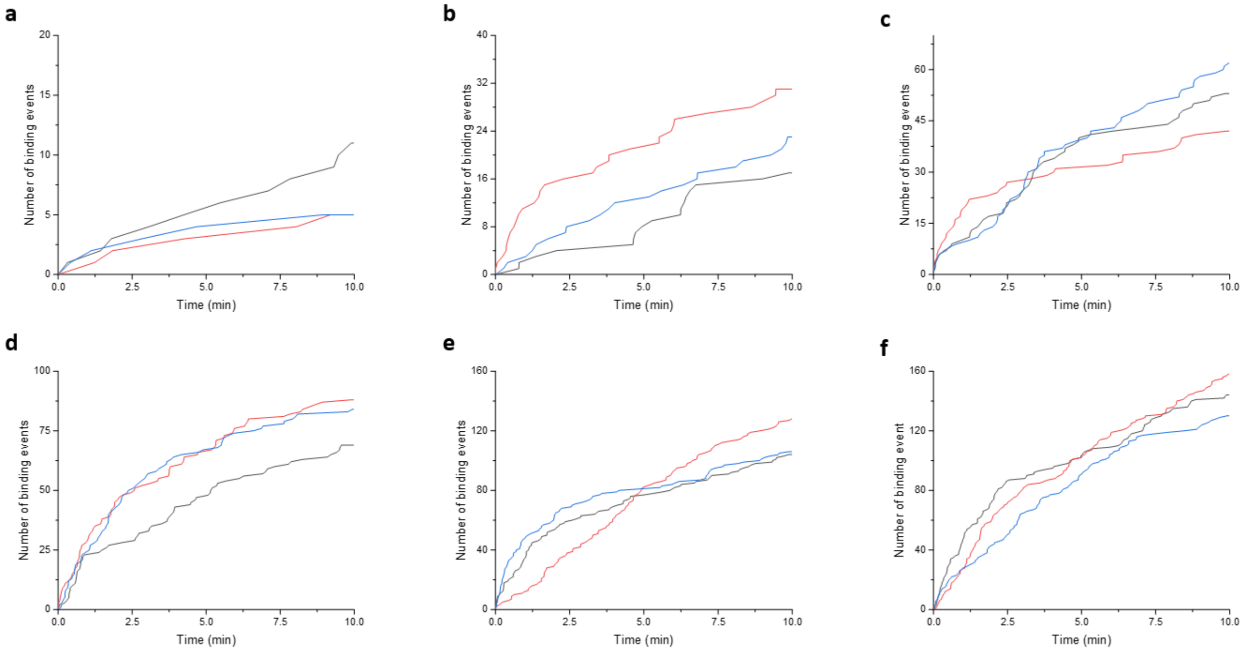

Figure S7. Raw calibration data of IL-6 detection in bovine whole blood. (a–f) The real-time counting results of IL-6 spiked bovine whole blood with concentrations of 0, 21 fg/mL, 210 fg/mL, 2.1 pg/ml, 21 pg/mL, and 210 pg/mL.

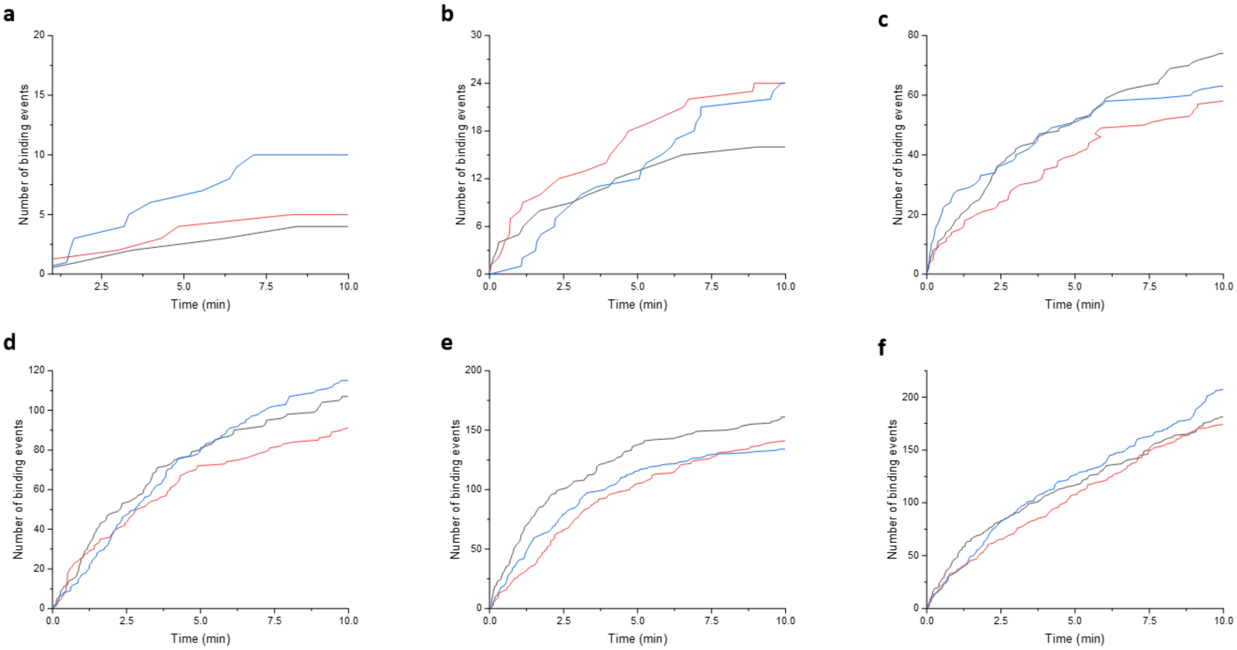

Figure S8. Raw calibration data of PSA detection in bovine whole blood. (a–f) The real-time counting results of PSA spiked bovine whole blood with concentrations of 0, 32 fg/mL, 320 fg/mL, 3.2 pg/ml, 32 pg/mL, and 320 pg/mL.

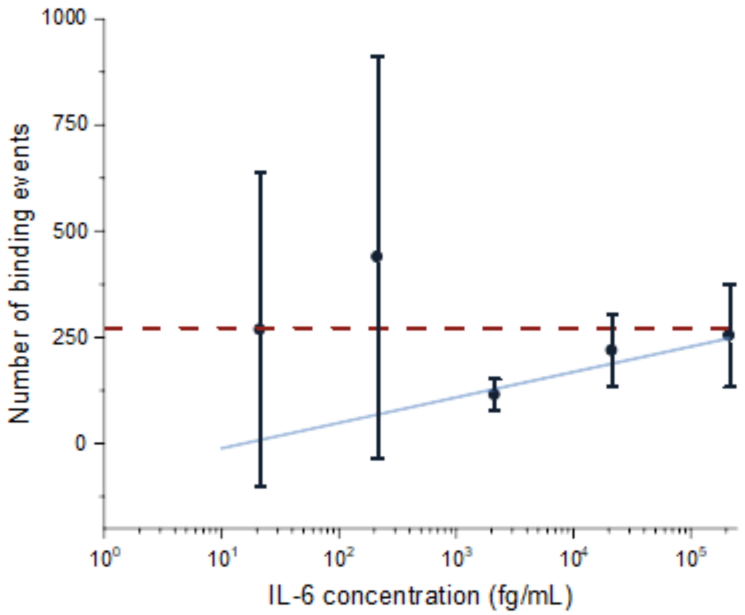

Figure S9. The standard curve for IL-6 detection in spiked bovine whole blood without passing through the binding event filters of our real-time counting algorithm. The dashed line indicates the limit of detection, defined as the mean plus three times s.d. of the counts of blank control without spiked analyte. (Details of all data points are shown in Figure S10)

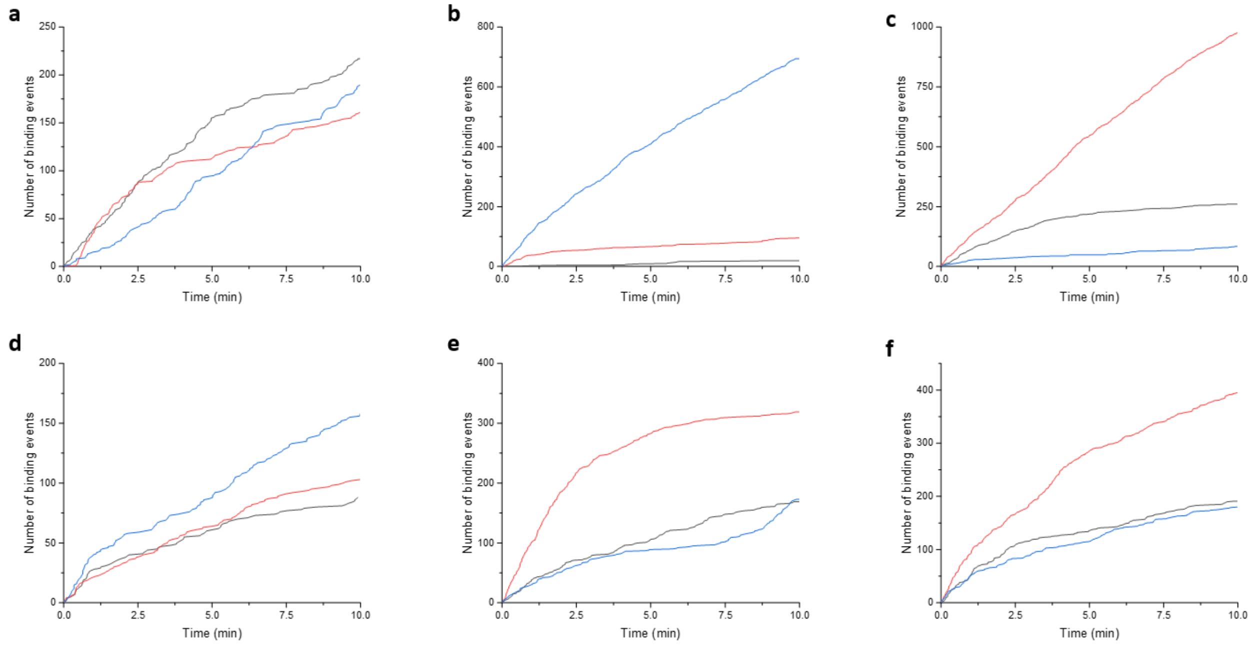

Figure S10. Total number of IL-6 binding events over time in spiked bovine whole blood before passing through the binding event filters. The concentrations of IL-6 from a to f are 0, 21 fg/mL, 210 fg/mL, 2.1 pg/ml, 21 pg/mL, and 210 pg/mL, respectively.

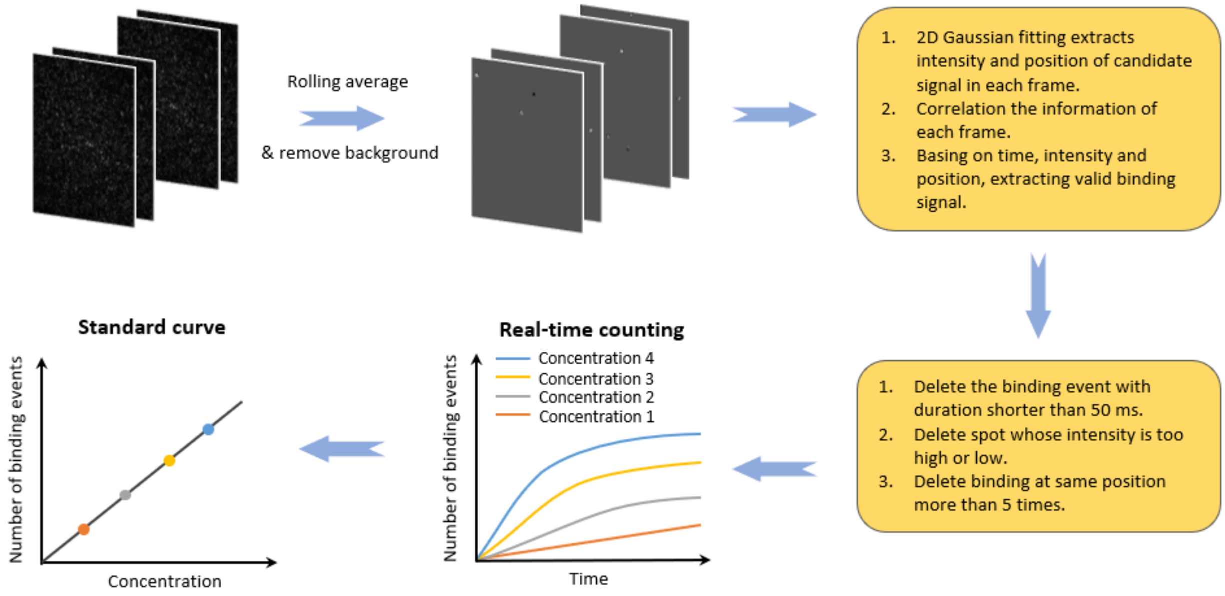

Figure S11. Automatic algorithm for counting the real-time specific binding events of the detection antibody. The raw image sequence was rolling averaged to remove the background noise. The denoised images were converted to probability images, from which candidate pixels of a binding event in a certain frame were identified. A Gaussian function was fitted to the pixels of the denoised images with the same coordinates as the identified candidate pixels. Based on the Gaussian fitting, invalid binding events were filtered out, and the position and intensity of valid binding events were determined. The detected binding events in all the frames were mapped over time to their spatial locations. Binding events from consecutive frames were treated as binding of an individual detection antibody if their coordinates were within diffraction limit distance from each other. Finally, the temporal profile of total binding events of the detection antibody was determined after nonspecific binding or surface impurities were filtered out.

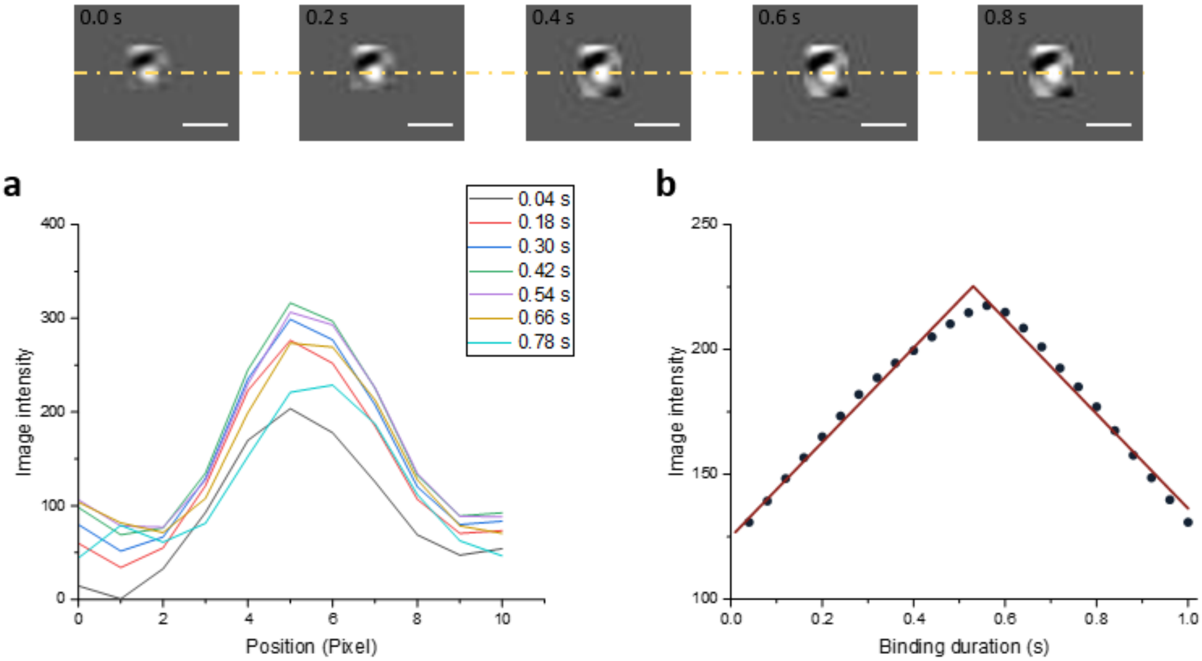

Figure S12. Extraction of a single protein binding event from the PSM image sequence. Representative images at different time points of a binding event were shown in the top panels (scale bars, 1 μm). (a) The corresponding image intensity profiles of the dashed lines are shown in the top panels. (b) Corresponding maximum intensities (dark dot) of the binding particle extracted by Gaussian fitting and linear fit to the intensity time course (red). The result is consistent with previous research, which shows that the binding intensity would grow and fade[1].

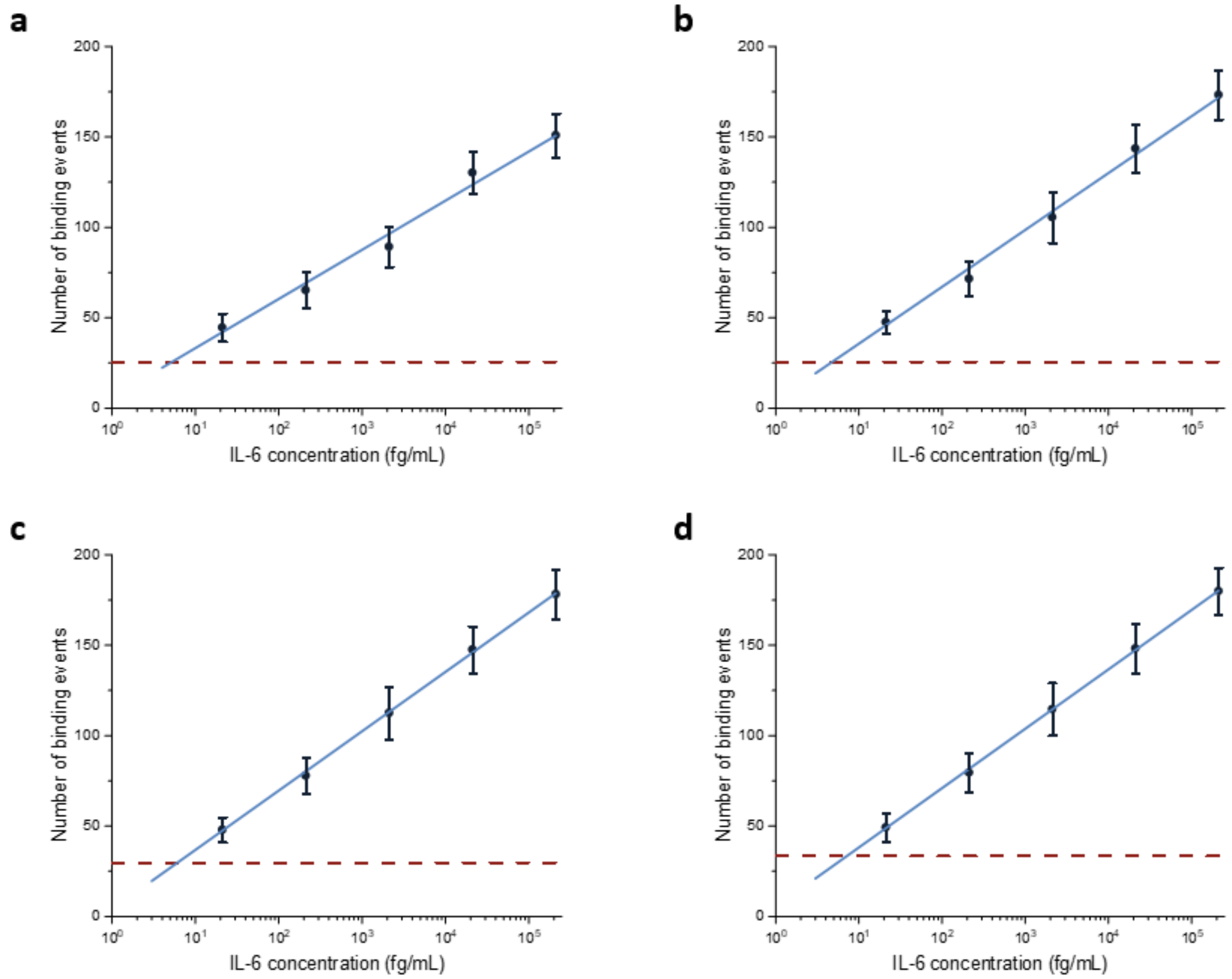

Figure S13. Standard curves of IL-6 detection in the pure buffer corresponding to different binding frequency thresholds. The thresholds used are (a) 1, (b) 2, (c) 5, and (d) 10 times in ten mins (#/10 mins), respectively. The dashed lines indicate the LOD (mean + 3×s.d. of the count of blank buffer). The error bars are s.d. of triplicate tests. The Details of each standard curve are shown in Table S1.

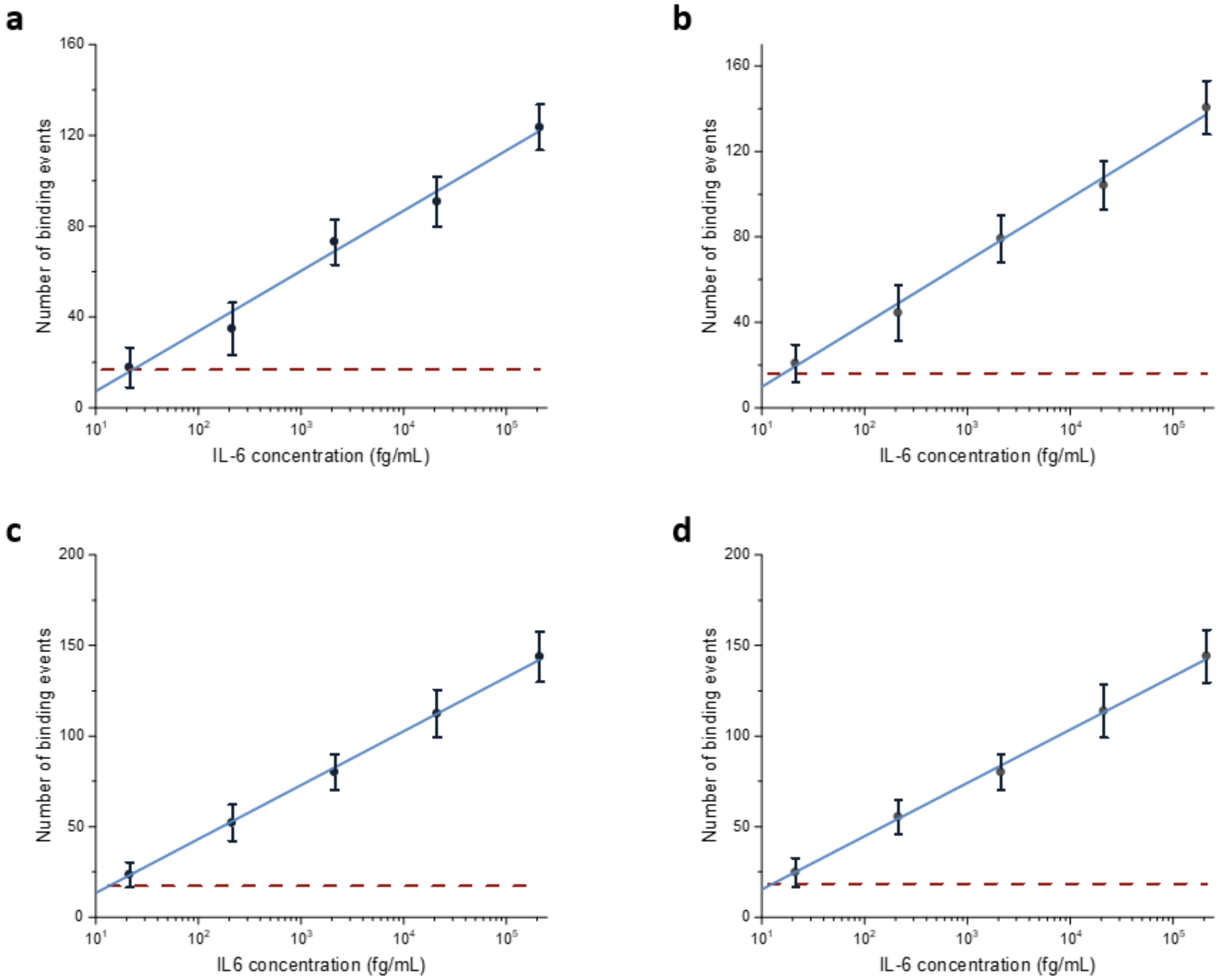

Figure S14. Standard curves of IL-6 detection in bovine whole blood with different binding frequency thresholds: (a) 1, (b) 2, (c) 5, and (d) 10 times in ten mins (Same set of thresholds as in Figure S13). Details of each standard curve are shown in Table S2.

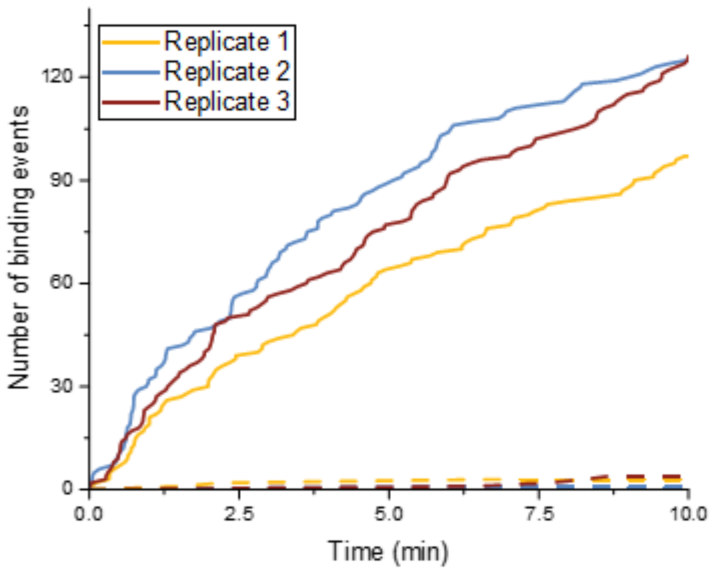

Figure S15. Real-time counting of binding and unbinding events. Time courses of detection antibody binding to IL-6 captured by the sensor surface represented as the counting of binding events (solid line) and unbinding events (dash line). The experiments were repeated three times under the same conditions.

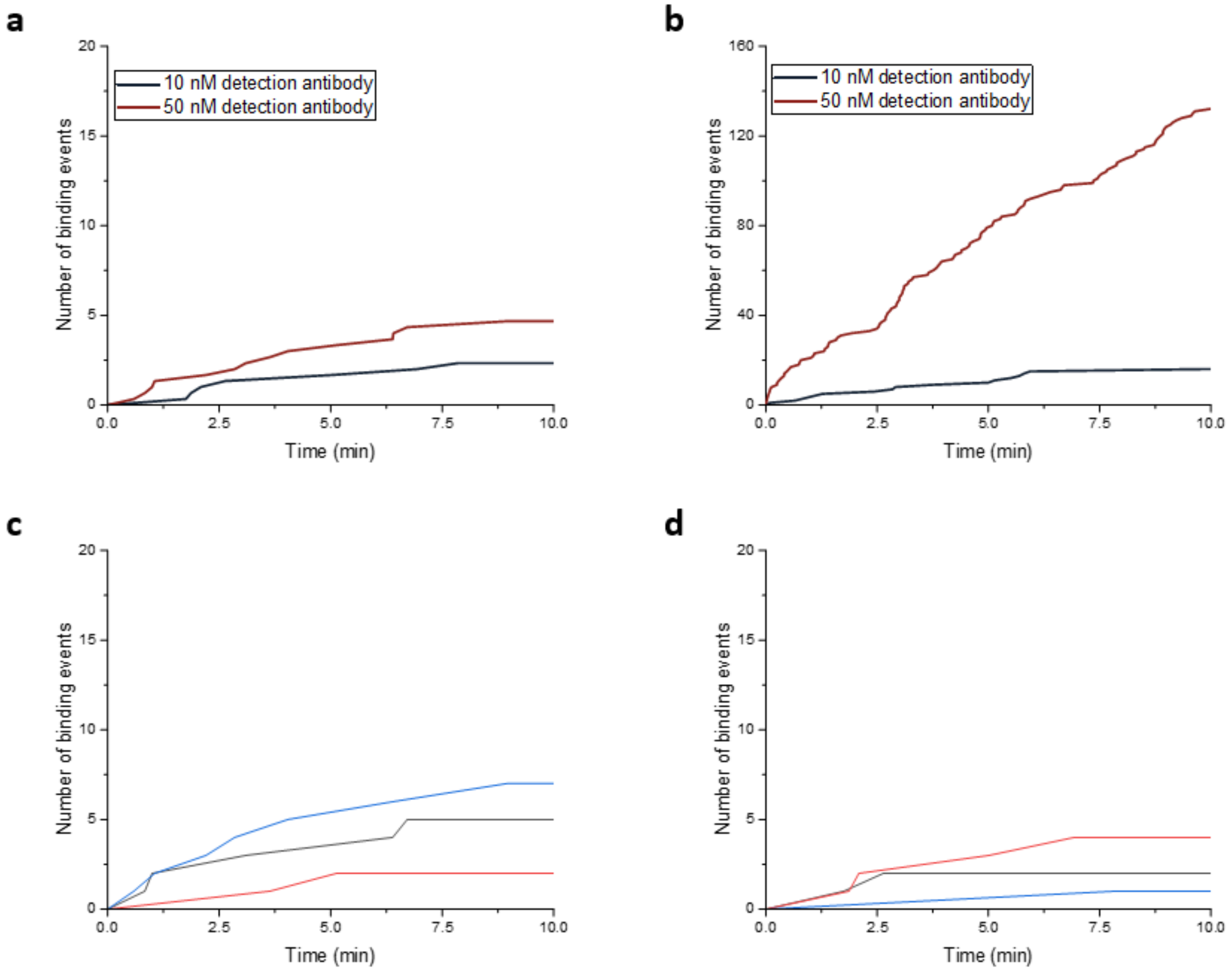

Figure S16. Influence of detection antibody's concentration on NT-proBNP detection. The number of binding events vs binding time of (a) blank buffer and (b)100 fM NT-proBNP in human plasma. For clarity, the averaged curves of triplicate tests of (c) 50 nM detection antibody and (d) 10 nM detection antibody were plotted in (a).

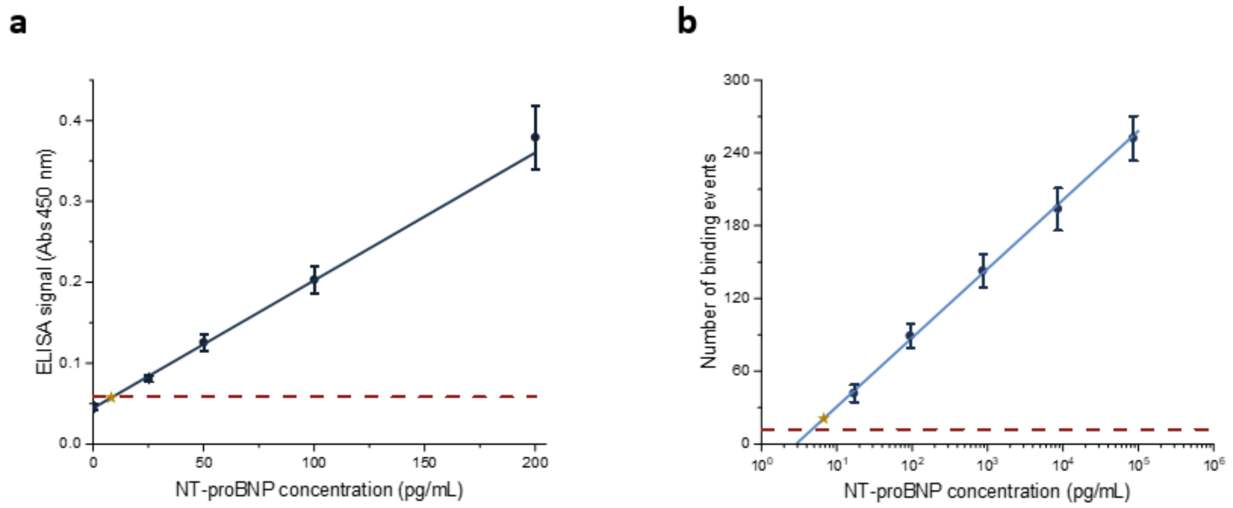

Figure S17. Investigation of the effect of endogenous NT-proBNP in human blood. (a) Conventional ELISA measurement of the endogenous NT-proBNP level in the normal human plasma used to generate LFSM-immunoassay standard curve. Recombinant NT-proBNP was spiked into horse serum with final concentrations of 25, 50, 100, and 200 pg/mL. The spiked horse serum standard solutions were measured by conventional ELISA and the corresponding absorbance readings were used to obtain the linear standard curve fit (r-square = 0.996). The dashed line is the LOD of NT-proBNP in horse serum by ELISA. The yellow star represents the measurement of endogenous NT-proBNP level. (b) LFSM-immunoassay measurement of the endogenous NT-proBNP level in the normal human plasma. The yellow star represents the endogenous NT-proBNP measurement on the LFSM-immunoassay standard curve.

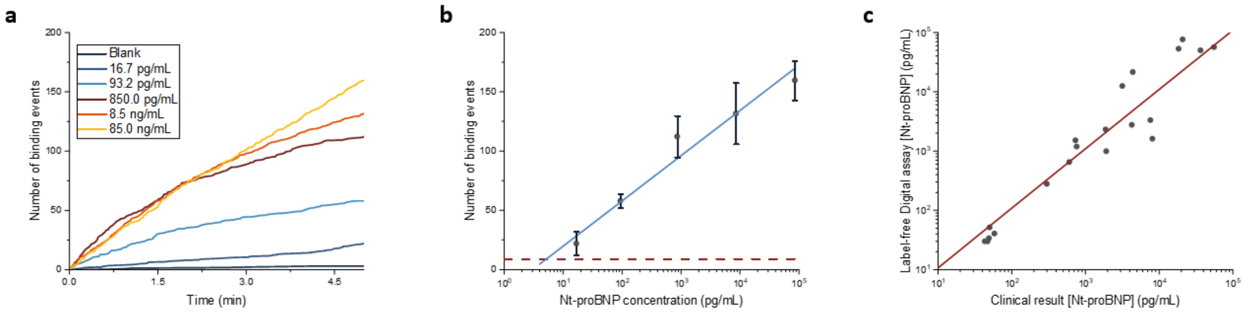

Figure S18. NT-proBNP detection in human plasma with 5-min counting time. The same set of raw data as in Figure 3 was processed in the same way to plot this figure, except that only the first 5 mins data was considered here. (a) 5-min temporal profiles for different NT-proBNP concentrations in human plasma. (b) Standard curve of NT-proBNP detection in human plasma for 5-min counting time. (c) Pearson's correlation between LFSM-immunoassay and Roche's Elecsys proBNP II assay using 5-min counting time.

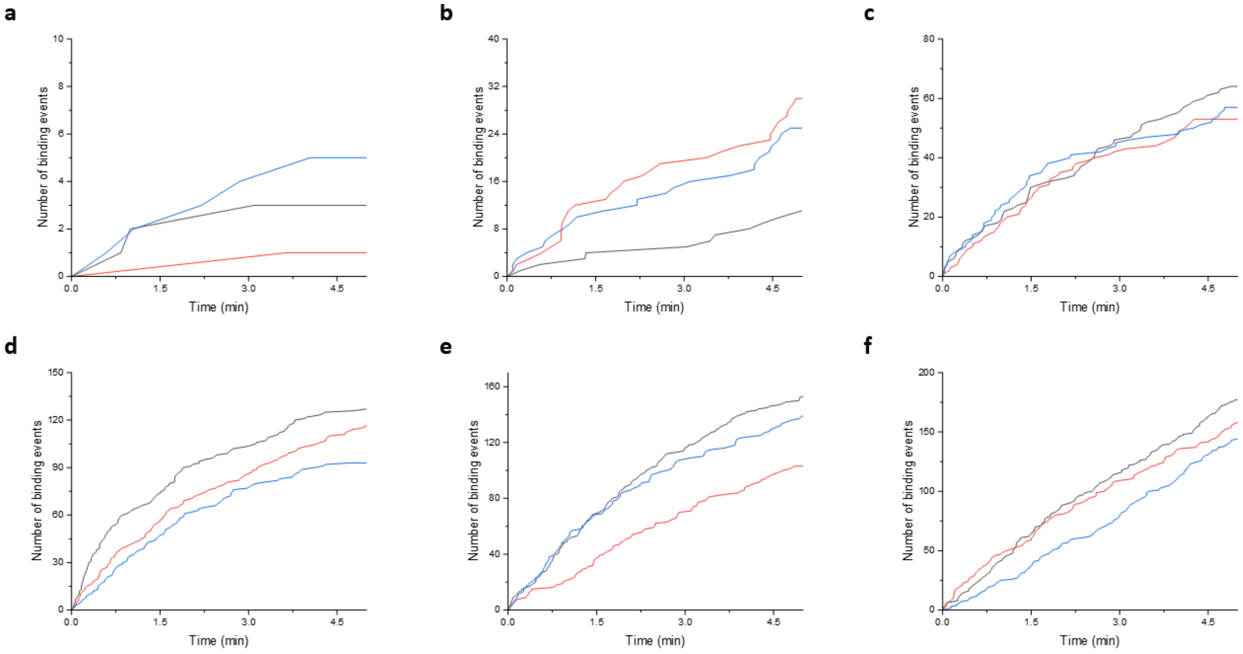

Figure S19. Raw 5-min temporal profiles of triplicate tests corresponding to (a) blank buffer (horse serum), NT-proBNP spiked human plasma with concentrations of (b) 16.7 pg/mL, (c) 93.2 pg/mL, (d) 850 pg/ml, (e) 8.5 ng/mL and (f) 85 ng/mL.

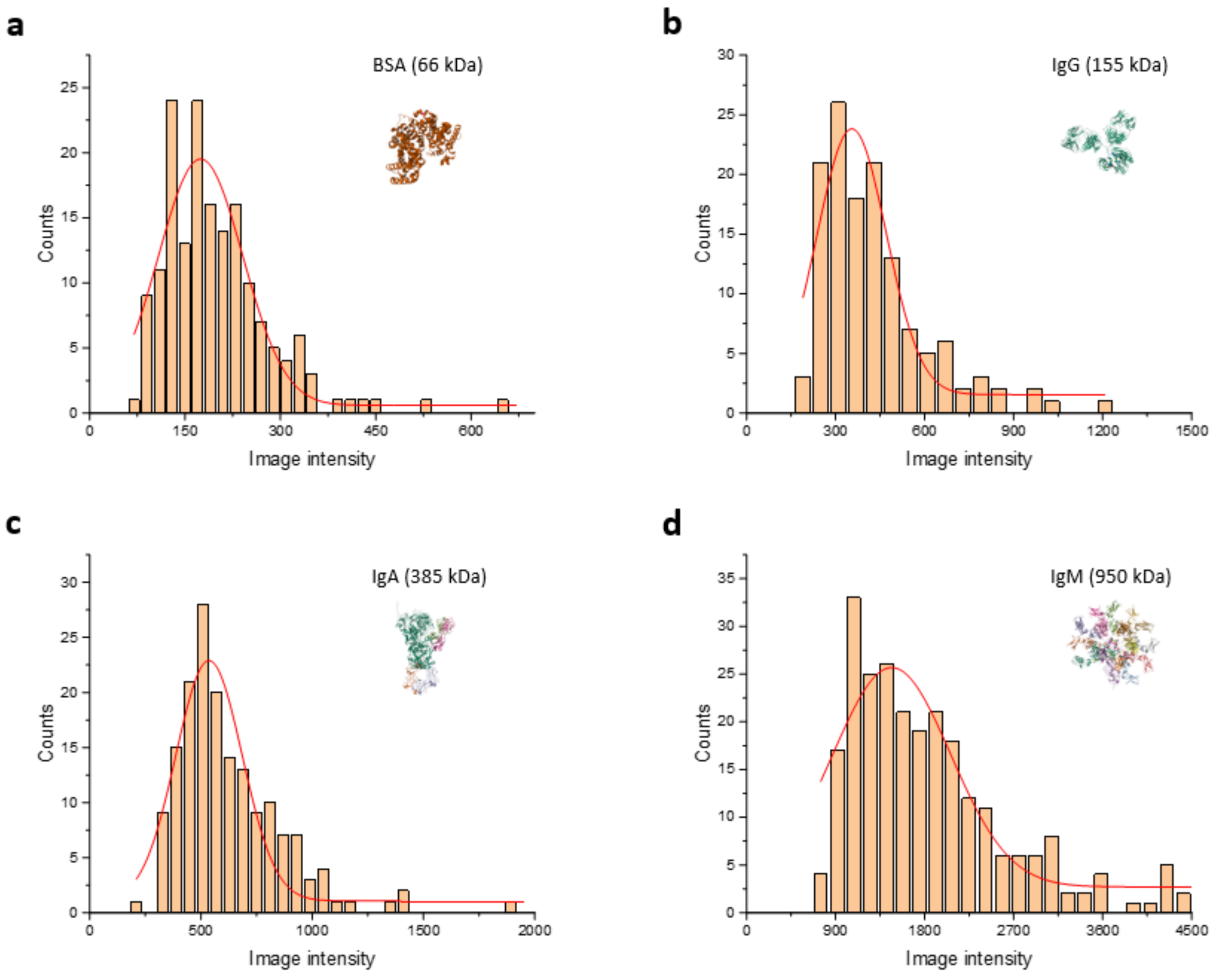

Figure S20. Calibration data of LFSMiA-lite mass detection. To determine the relationship between image intensity and protein molecular weight, 4 different proteins with known molecular weight were dissolved in PBS buffer and flowed over the bare gold surface. Binding events of different proteins were recorded and extracted to obtain histograms of their image intensity. The solid lines are the Gaussian fitting results for the 4 proteins. The incident light intensity was 2 kW/cm^2. The exposure time was 2.5 ms for IgM, 5 ms for IgA, 10 ms for IgG, 15 ms for BSA. All the results were normalized to exposure time of 15 ms.

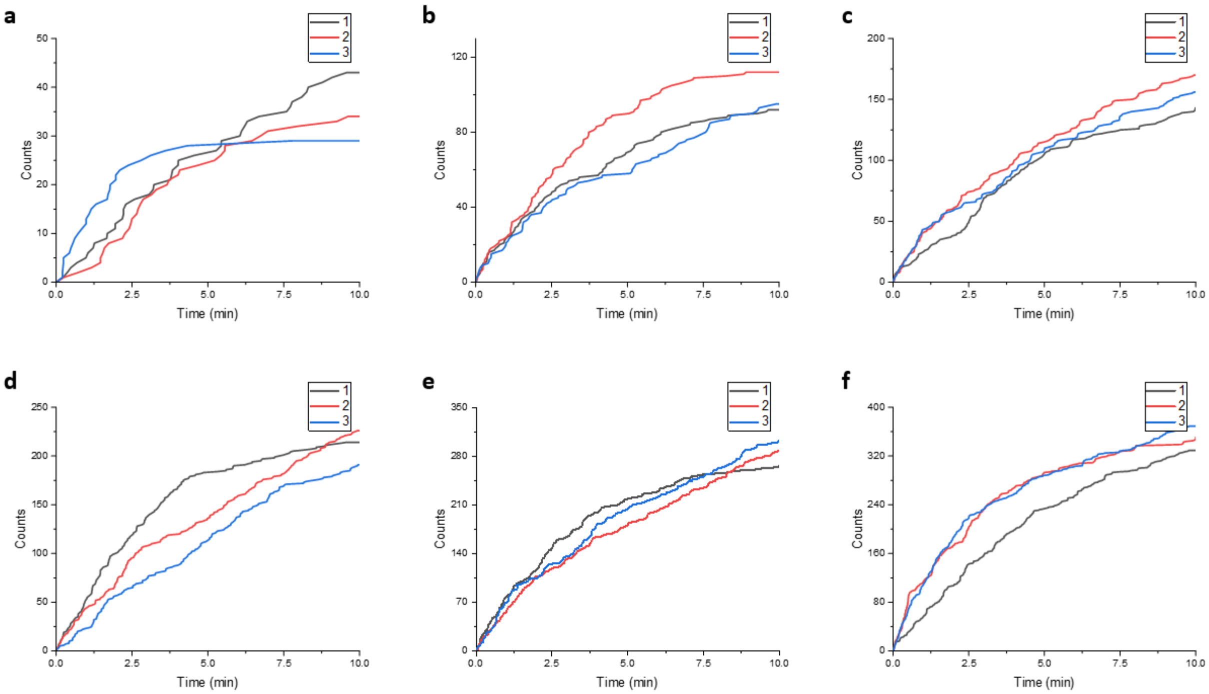

Figure S21. Raw calibration data of IL-6 detection in bovine whole blood on LFSMiA-lite. (a–f) The real-time counting results of IL-6 spiked bovine whole blood with concentrations of 0, 21 fg/mL, 210 fg/mL, 2.1 pg/ml, 21 pg/mL, and 210 pg/mL.

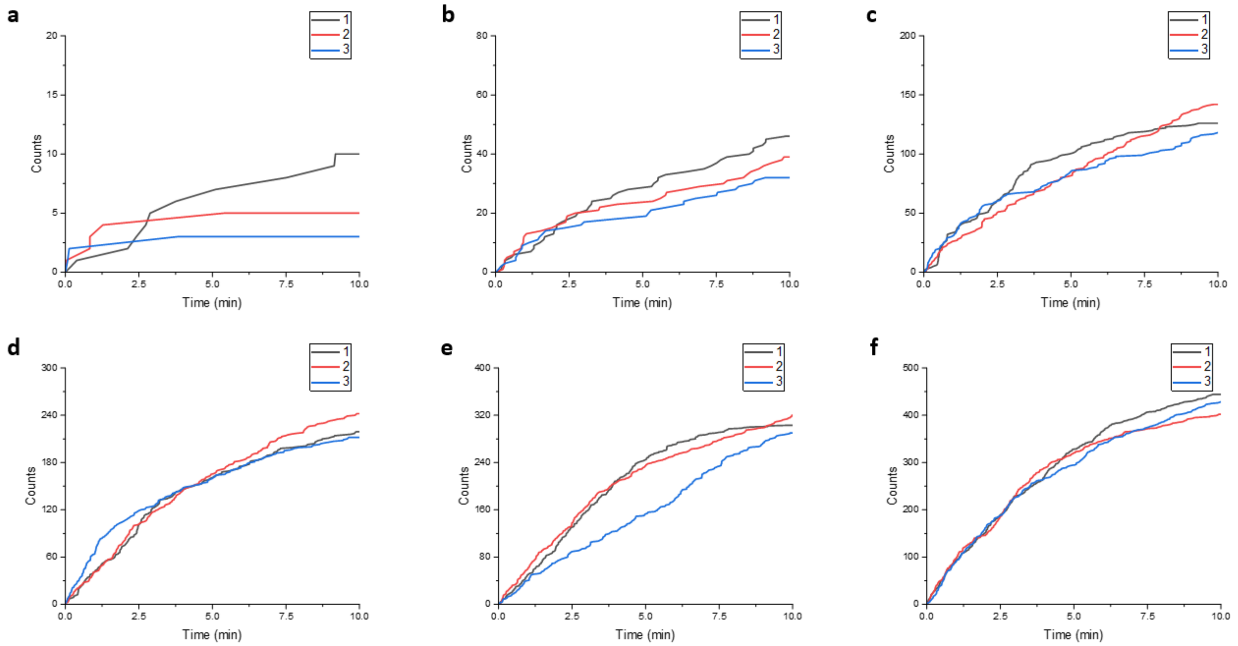

Figure S22. Real-time counting results of total binding events on LFSMiA-lite for generating NT-proBNP standard curve corresponding to (a) blank buffer (bovine whole blood), NT-proBNP spiked bovine whole blood with concentrations of (b) 8.5 pg/mL, (c) 85.0 pg/mL, (d) 850 pg/ml, (e) 8.5 ng/mL and (f) 85 ng/mL.

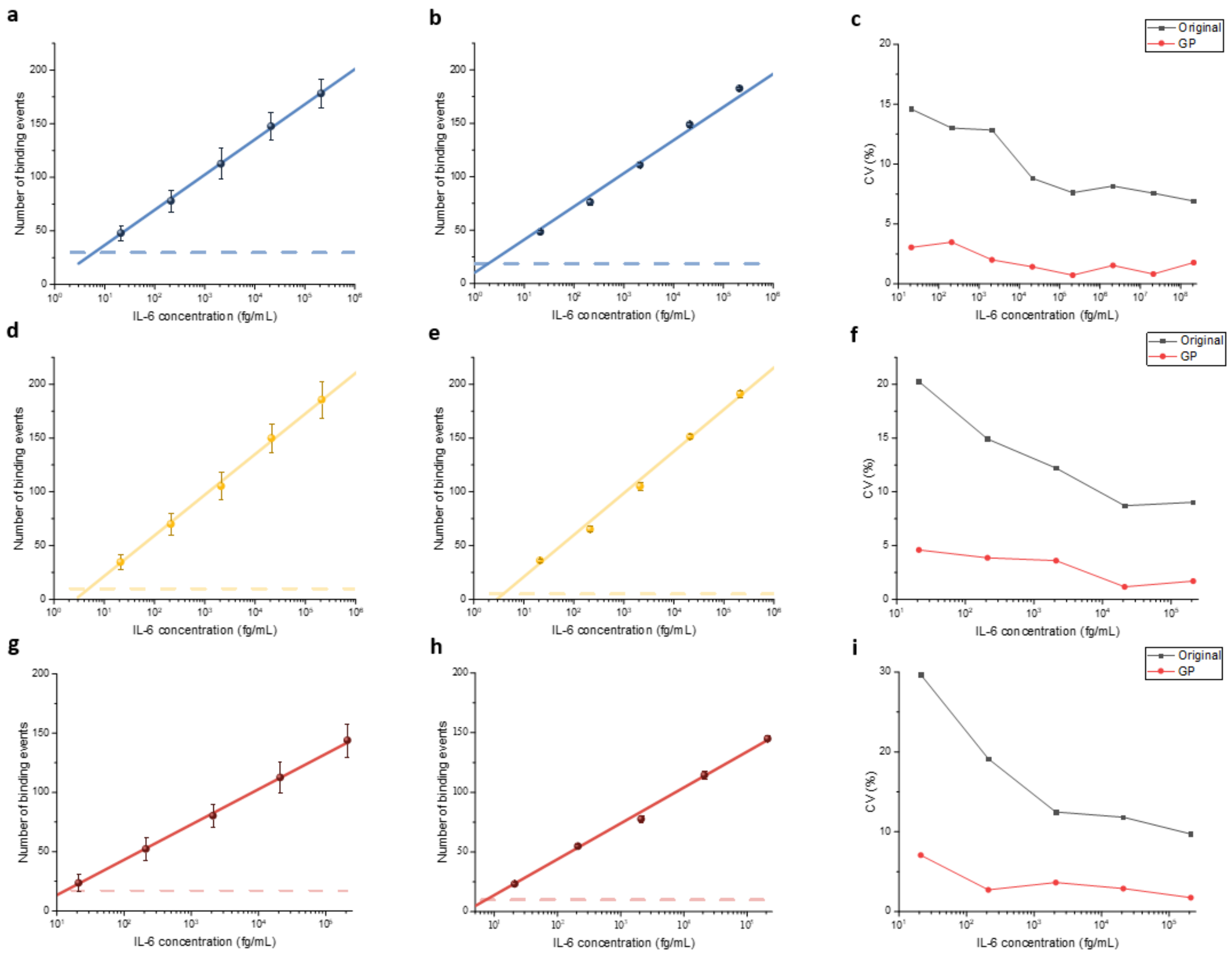

Figure S23. Improvements of Gaussian process model. (a), (d) and (g) are the standard curves of IL-6 detection in pure buffer, horse serum, and bovine whole blood determined by the total counts in 10 mins, respectively. (b), (e) and (h) are the corresponding standard curves processed by GP model. (c), (f) and (i) show the improvements of the GP model on the CV of IL-6 detection in pure buffer, horse serum and bovine whole blood, respectively.

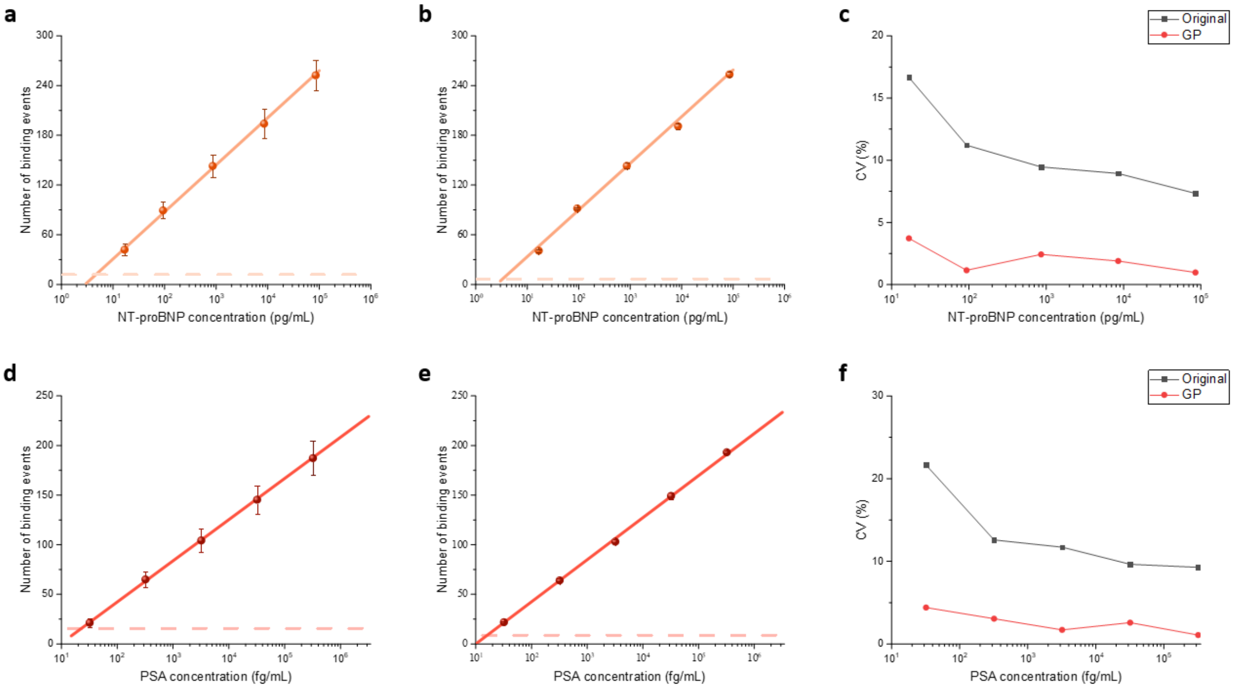

Figure S24. Improvements of Gaussian process model. (a) and (d) are the standard curves of NT-proBNP detection in human plasma and PSA detection in bovine whole blood determined by the total counts in 10 mins. (b) and (e) are the corresponding standard curves processed by the GP model. (c) and (f) show the improvements of the GP model on the CV of NT-proBNP detection in human plasma and PSA detection in bovine whole blood, respectively.

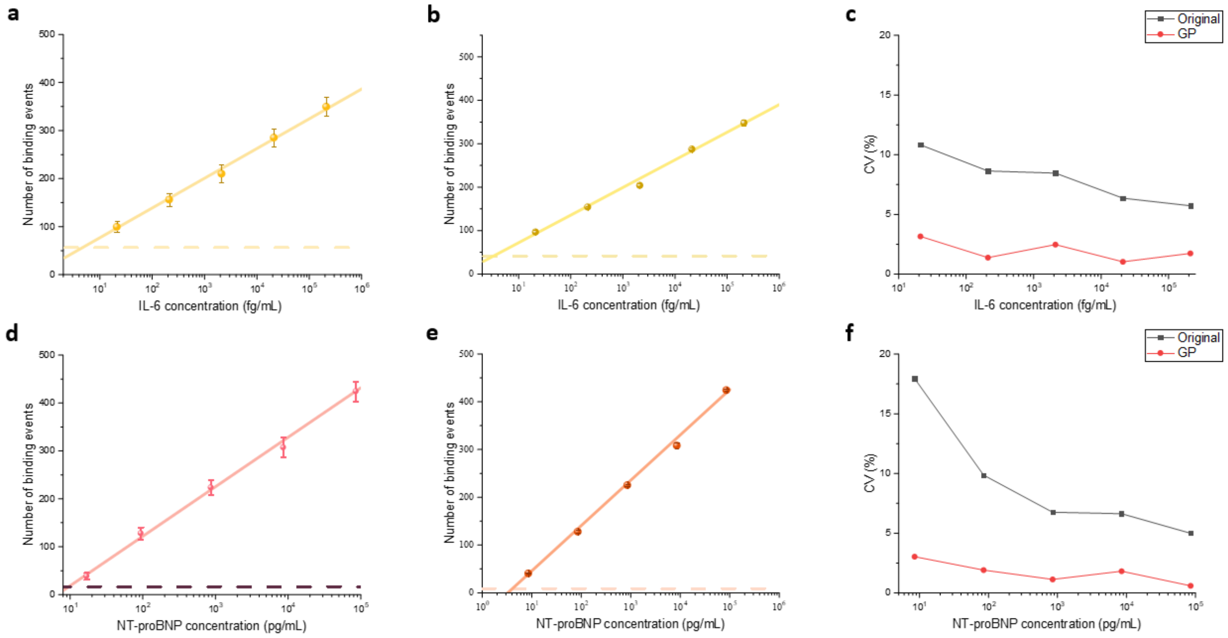

Figure S25. Improvements of Gaussian process model. (a) and (d) are the standard curves of IL-6 detection in horse serum and NT-proBNP detection in bovine whole blood on LFSMiA-lite determined by the total counts in 10 mins. (b) and (e) are the corresponding standard curves processed by the GP model. (c) and (f) show the improvements of the GP model on the CV of IL-6 detection in horse serum and NT-proBNP detection in bovine whole blood on LFSMiA-lite, respectively.

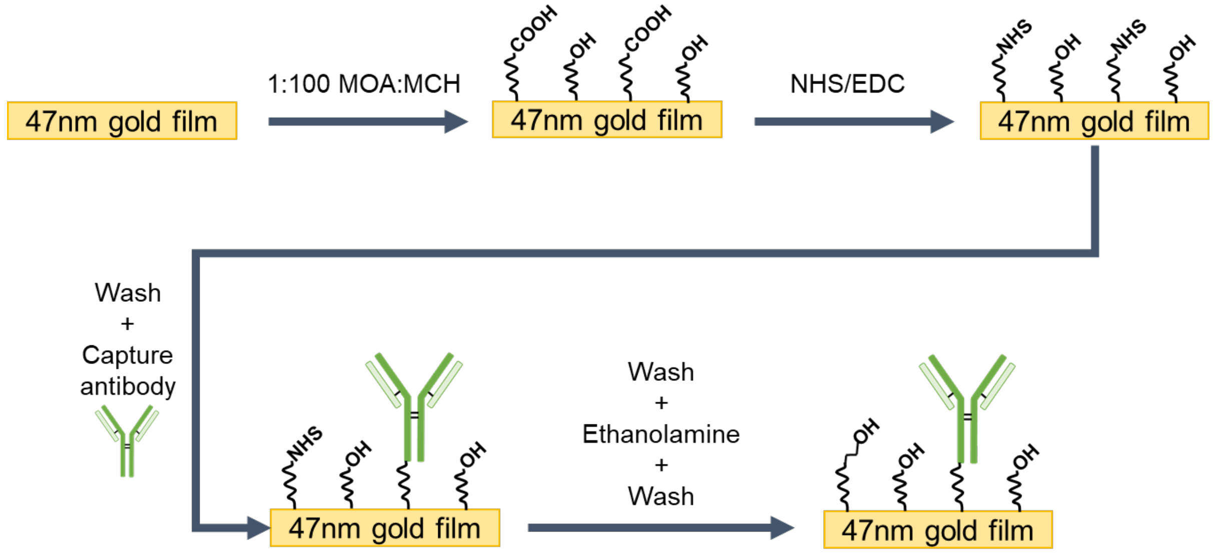

Figure S26. Sensor surface functionalization. The gold substrate was first dipped in an ethanol solution with 100 µM 8-mercaptooctanoic acid (MOA) and 10 mM 6-mercapto-1-hexanol (MCH) overnight. After being rinsed with ethanol and DI water three times, 60 µL EDC and NHS mixed solution (5 mM and 10 mM in DI water) was injected 3 times into the microfluidic channel at 0, 5, and 10 min in a 15-min total incubation time. The sensor was then cleaned with 200 µL MOPS buffer. Capture antibody with a concentration of 100 µg/mL in MOPS buffer was injected three times to improve the efficiency of surface modification. The capture antibody coated sensor was then quenched with 1 M ethanolamine at pH 9.6.

Tables S1

The limit of detection (LOD), r-square (COD), and coefficient of variance (CV) of the standard curves of IL-6 detection in the buffer for different binding frequency thresholds (times in 10 mins) as shown in Figure S13.

Threshold	LOD	COD	CV
1	5.38 pg/mL	0.98224	12.46 %
2	4.88 pg/mL	0.99251	11.67 %
5	6.18 pg/mL	0.99926	11.37 %
10	7.31 pg/mL	0.99941	11.76 %

Tables S2

The limit of detection (LOD), r-square (COD), and coefficient of variance (CV) of the standard curves of IL-6 detection in bovine whole blood for different binding frequency thresholds (times in 10 mins) as shown in Figure S14.

Threshold	LOD	COD	CV
1	23.22 pg/mL	0.98503	23.08 %
2	16.70 pg/mL	0.99445	20.86 %
5	13.60 pg/mL	0.99873	16.57 %
10	13.18 pg/mL	0.99703	16.83 %

Tables S3

Comparison with existing POC NT-proBNP Assays.

Name	Methods	Biomarker	Specimen	Sample size	Detection limit (pg/mL)	Assay time
Quantum dots	Later flow assay	NT-proBNP	serum	25 µL	40	20 minutes
*oc*FLOW	Later flow assay	NT-proBNP	serum	20 µL	6375	15 minutes
SPRWR	SPR	NT-proBNP	plasma	25 µL	1300	20 minutes
Dry-reagent microfluidic sensor	Electrochemical	NT-proBNP	serum	10 µL	570	15 minutes
HEMT	Field effect transistor	NT-proBNP	plasm	4 µL	50	23 minutes
i-STAT	Sandwich immunoassay	BNP	Whole blood	17 µL	15	10 minutes
Pathfast	Chemiluminescent	NT-proBNP	Whole blood	100 µL	15	17 minutes
AQT90 FLEX	Fluorescence	NT-proBNP	Whole blood	> 1 mL	20	11 minutes
Ramp 200	Fluorescence	NT-proBNP	Whole blood	75 µL	18	15 minutes
Cobas h232	Later flow assay	NT-proBNP	Whole blood	150 µL	60	12 minutes
Stratus CS	Fluorescence	NT-proBNP	Whole blood	≥ 3 mL	15	14 minutes
LumiraDx	Fluorescence	NT-proBNP	Whole blood	20 µL	50	12 minutes
LFSMiA	Plasmonic scattering	NT-proBNP	Whole blood	40 µL	3.39	20 minutes

Note S1. Binding event detection.

The binding event was extracted following the procedure shown in Figure S11. The noise in the image was first reduced by applying 25 frames rolling averaging of the raw video starting at frame m, N_m^{m+25}, and then normalizing each frame of the averaged video in terms of their mean pixel value to avoid the intensity change from the light source. A differential image sequence was obtained by subtracting each normalized frame from its subsequent frame ($N_{m+25}^{m+50} - N_m^{m+25}$), which removed the rough background and revealed the binding and unbinding events. The differential images were then convoluted with Haar-like arrays to be transferred into a probability image (PI), which extracted the morphological characteristics in the image[2]. Pixels with intensity higher than the mean intensity + 3 × standard deviation (s.d.) of the whole PI were selected as candidate pixels. For each pixel of the differential image with the same coordinates as the candidate pixels of the PI, a fixed neighborhood (11×11 pixel^2) was extracted and fitted with a 2D Gaussian function to get the precise center location and intensity of the particle. The 2D Gaussian model is defined as:

$$f(x, y) = Ae^{\left[\frac{(x-x_0)^2}{2\sigma_x^2} - \frac{(y-y_0)^2}{2\sigma_y^2}\right]} + c \qquad [S1]$$

where (x_0, y_0) is the position of the binding event in the differential image, (σ_x, σ_y) are standard deviations of the 2D Gaussian model, which contains the morphological information of the binding particle, A is the intensity of the binding particle, and c is the offset constant. The pixels would be rejected if the ratio between σ_x and σ_y was smaller than 0.7 or larger than 1.43 as the binding particle is not a single molecule[1]. The binding event of a single particle appears as a brightening Gaussian blob and then gradually disappears in several consecutive frames (Figure S12). To avoid over-counting particles, binding events from consecutive frames whose center positions fall within a fixed distance ($|x_1 - x_2| + |y_1 - y_2| \leq 3$ pixels) would be grouped as one binding event. To extract the most accurate image intensity of the binding event, a function I(t), whose value would grow linearly up to a maximum and then decrease linearly, was fitted to the profile of the binding intensity VS time and the maximum value of the fitted model was chosen as the intensity of the binding event. I(t) is defined as:

$$I(t) = M - |k(t - t_0)| \qquad [S2]$$

Where I is imaging intensity, M is the accurate intensity of the binding event, k is a constant representing the speed of the binding event, and t_0 is the accurate time the particle hits the surface. The binding event that did not appear in 5 continuous frames would be considered as a nonspecific binding and deleted. Based on the relationship between MW and image intensity (Figure S2)[3,4], the detected binding events would be considered as detection antibody binding to the analyte if their image intensity were within the range of 60 to 240 (consider the formation of dimers).

Note S2. Determination of the threshold for binding time.

As the clinical sample contains proteins of various sizes, label-free imaging could not confidently distinguish the dissociation event of the detection antibody and that of protein with a similar size after the sensor was incubated in a complex sample. We analyzed the binding of IL-6 detection antibody to surface captured IL-6 (Figure S15). After immobilizing the capture antibody, BSA solution was used to passivate the surface. 100 fM IL-6 in buffer was flowed over the sensor surface for 1 hour. Then 3 mL of 10 nM IL-6 detection antibody was introduced into the system for 10 mins, which was recorded by PSM imaging. Similar to the algorithm for detecting binding events, the only difference in detecting dissociation events was that only the dissociation events with an image intensity in the range of -60 to -240 were considered detection antibodies leaving the surface. As shown in Figure S15, the number of dissociation events can be ignored compared with the number of binding events. Therefore, we only counted the binding event in our assay time.

As shown in the support video, the main difference between nonspecific binding and specific binding is that the nonspecific binding would hit the same place on the sensor surface more times, assuming most nonspecific binding events are weak in affinity. To estimate the probability of two detection antibodies binding to the same area on the surface, we can assume that the surface is composed of 1000 blocks with evenly distributed antigen molecules. So, the probability of a detection antibody binding to a certain block is 0.001. The probability of the event where n detection antibodies in a total of 100 detection antibodies bind to the same block can be calculated as

$$p(n, 100) = C_{100}^{n} 0.001^{n} 0.999^{100-n}$$

The probabilities for n = 0, 1, and 2 are 0.9048, 0.0906, and 0.0045 respectively. Therefore, the probability of several detection antibodies binding to the same position is low, and a threshold of binding frequency can be used to distinguish specific binding and nonspecific binding. We used different thresholds of binding time to process the data of IL-6 detection in pure buffer and whole blood. The standard curves are shown in Figures S13 and S14. The details of the standard curves of pure buffer and whole blood are shown in tables S1 and S2, respectively. Comparing the LOD, COD, and CV of different thresholds, the threshold value of “5 times in 10 minutes” has at least two-thirds of the parameters better than the other threshold values in both pure buffer and whole blood groups.

Note S3. Bayesian Gaussian process model for the binding event.

From a statistical perspective, we model the real-time binding event with a stochastic process indexed by time, and the measured binding counts are viewed as observations of the underlying binding process at the designated time points. As the binding process is continuous over time and contains the dependence between count values at any two time points, a Gaussian process becomes a natural fit for the binding process. Gaussian process modeling is a famous supervised machine learning method due to its flexibility in generating smooth realizations. The associated covariance kernel function controls the dependence among function values, which allows the estimation to borrow information from the neighborhood. In particular, we implemented the Gaussian process

model under a Bayesian framework to not only obtain the binding count estimates but also provide quantification of uncertainties (posterior standard deviation) in the obtained estimates.

Gaussian process modeling of the binding events. We describe the binding process as a continuous real-valued function of time, defined as $f : \mathcal{T} \to \mathcal{R}$, a mapping from the time interval $\mathcal{T}$ of interest to the real line $\mathcal{R}$. We assume that the binding count function follows a zero-mean Gaussian process $f : \mathcal{T} \to \mathcal{R}$ as $f \sim \mathrm{GP}(0, \mathrm{c}(\cdot, \cdot))$ with a bivariate covariance kernel function $\mathrm{c}(\cdot, \cdot) : \mathcal{T} \times \mathcal{T} \to \mathcal{R}^+$, here $\mathcal{R}^+$ denotes the positive part of the real line. The covariance kernel function only produces positive correlations by default. The type of covariance kernel function determines the correlation level between the binding counts measured at two different time points, which will be chosen later. As there is no evidence that the binding process follows a certain trend, we set the mean of the Gaussian process to be constant zero over time. By the definition of a Gaussian process, at the designated time points $\{t_1, \cdots, t_n\}$, the evaluated binding counts $[f(t_1), \cdots, f(t_n)]$ jointly have a n-dimensional multivariate normal distribution as

$$f_n := \begin{bmatrix} f(t_1) \\ f(t_2) \\ \vdots \\ f(t_n) \end{bmatrix} \sim N(0_n, \mathcal{T}_n), \qquad [S3]$$

where the mean vector is an n-dimensional zero vector 0_n and the variance-covariance matrix $\mathcal{T}_n$ is a $\mathrm{n} \times \mathrm{n}$ symmetric and positive definite matrix with its (i, j)-th element $\mathcal{T}_{i,j} = \mathrm{c}(t_i, t_j)$ for $1 \leq i, j \leq n$. As the binding process is expected to be relatively smooth, we consider a squared-exponential covariance kernel function of the form

$$c_{SE}(x, y; \ell) = exp\left\{-\frac{(x-y)^2}{2\ell^2}\right\}, \text{for any } x, y \in \mathcal{T}, \qquad [S4]$$

where $\ell > 0$ is the associated length-scale parameter that controls the correlation level between the function evaluations at different locations. The squared-exponential kernel function is commonly used to generate smooth GP realizations, which contain strong dependence. Implementing the squared-exponential kernel in Equation S3 yields that $f_n \sim N(0_n, \mathcal{T}_\ell)$, where $\mathcal{T}_\ell$ is the correlation matrix with its (i, j)-th element $\mathcal{T}_{i,j} = c_{SE}(x, y; \ell)$ for any $1 \leq i, j \leq n$.

Bayesian inference. The main goal is to recover the binding counts from the noised observations as well as to quantify the uncertainty in the estimates. We specifically take a Bayesian approach for estimation due to its advantage in uncertainty control. Given the data Y, one may define a likelihood function $P_\theta(Y)$ that links the data and the assumed model associated with an unknown parameter θ to be estimated. Under a Bayesian framework, one may treat the unknown parameter θ as random quantities that are endowed with some distribution $\Pi(\theta)$, named as the prior distribution. The prior distribution is determined to incorporate the *a priori* knowledge on the parameter. Bayesian inference is conducted through the posterior distribution which combines the prior information and the likelihood. That being said, the posterior distribution is obtained by applying the Bayes' rule,

$$\Pi(\theta|Y) = \frac{P_\theta(Y)\Pi(\theta)}{\int P_\theta(Y)\Pi(\theta)d\theta} \qquad [S5]$$

The posterior distribution $\Pi(\theta|Y)$ is a well-defined distribution of the parameter θ conditioning on the data Y. As follows, we will specify the likelihood function that links the binding count observations and the Gaussian process model. The prior distributions of the associated unknown parameters shall be provided as follows.

Gaussian process hierarchical model. We denote the collected data points $\{y_i, i = 1, \cdots, n\}$ of size *n* are noisy observations of the binding process at given time points $\{t_i, i = 1, \cdots, n\}$. We also assume that the measurement error, systematic error, and other unknown errors are random and additive to the true binding count values. Under the above assumptions, we now propose our Bayesian hierarchical model

$$y_i = f(t_i) + \varepsilon_i, \qquad \varepsilon_i \sim N(0, \sigma^2), \qquad [S6]$$

$$[f(t_1), \cdots, f(t_n)] \sim N(0_n,\ t^2\mathcal{T}_\ell), \qquad [S7]$$

$$\sigma^2 \sim \mathrm{IG}(a_\sigma, a_\sigma), t^2 \sim \mathrm{IG}(a_t, a_t), \ell \sim \mathrm{Unif}(a_\ell, b_\ell). \qquad [S8]$$

Equation S6 describes the likelihood function that links the observations $\{y_i, i = 1, \cdots, n\}$ to the assumed Gaussian process values $\{f(t_i), i = 1, \cdots, n\}$ evaluated at time $\{t_i, i = 1, \cdots, n\}$. We assume that the additive unknown random errors ε_i's are independently and identically distributed (*i.i.d.*) with a centered normal distribution with some positive variance parameter σ^2. The variance σ^2 is assumed to be unknown and is assigned with an Inverse-Gamma (IG) prior. As the binding function f is modeled with a Gaussian process associated with a squared-exponential covariance kernel function, Equation S3 implies a multivariate normal prior distribution for $[f(t_1), \cdots, f(t_n)]$ defined in Equation S7, where the correlation matrix $\mathcal{T}_\ell$ is defined in Equation S4, and the associated length-scale parameter ℓ is also assumed to be unknown. We consider a Uniform prior on ℓ. In addition, in Equation S7, the prior covariance matrix contains a positive scale parameter t^2 to control the magnitude of the variances, as the matrix $\mathcal{T}_\ell$ only controls correlations. The scale t^2 is unknown and is endowed with an Inverse-Gamma prior. More details on prior distributions are discussed in the hyperparameter specification section.

Hyperparameter specification. To simplify the fitting process, we standardize the observed binding counts to ensure that the observations are on the same scale in different cases, and also scale the time points to be all within [0, 1]. Specifically, for the error variance parameter σ^2, we specify that $\sigma^2 \sim \mathrm{IG}(a_\sigma, a_\sigma)$ with $a_\sigma = 0.01$. This prior is considered weakly informative[6] in the sense that the prior does not enforce a strong subjective preference on the value of σ^2, thus the posterior lets the data to update σ^2. The standardized binding counts in different cases range mainly in [-2, 2] with a unit standard deviation. A proper prior on f_n shall assign the major amount of the prior probability on the values of f_n which roughly are the same scale as standardized binding counts. It is easy to see that the values of f_n depend on the scale of t, the prior standard deviation. Therefore, the prior distribution of t^2 is determined such that f_n is also on the same scale as the standardized binding counts. Based on this discussion, we consider $t^2 \sim \mathrm{IG}(a_t, a_t)$

with $a_t = 5$ so that the prior mean is 1.25 and the prior standard deviation is approximately 0.7, also, there is around 0.9 prior probability that the prior standard deviation of f_n is less than 1.5. Our empirical results indicate that the performance of the model is not sensitive to the choice of a_t provided $a_t \in (2, 10)$. At least, for the length-scale parameter ℓ associated with the Gaussian process, we consider the prior choice $\ell \sim \text{Unif}(a_\ell, b_\ell)$ with $a_\ell = 0.1$, $b_\ell = 2$. This setting allows for the correlation between the two furthest time points in [0, 1] varying in $(5 * 10^{-5}, 0.6)$, allowing both a weak and strong long-range dependence possibly in the covariance matrix. The Uniform prior distribution is also noninformative so that the posterior distribution lets the data update the parameter ℓ.

Posterior Inference. For simplicity of notation, we rewrite the likelihood function in Equation S6 in a matrix form. Denoted all of observed binding counts by $Y = [y_1, \cdots, y_n]$ at the time points $T = [t_1, \cdots, t_n]$, then the likelihood function $P_{f_n}(Y) = N(Y; f_n, \sigma^2 I_n)$, we denote by I_n the identity matrix of dimension n. Here we use $N(Y; f_n, \sigma^2 I_n)$ to denote the normal distribution of Y with the mean vector (f_n) and the variance-covariance matrix $(\sigma^2 I_n)$. Applying Bayes' rule to the hierarchical model in Equation S6 – S8 yields the joint posterior distribution

$$\Pi[f_n, \sigma^2, t^2, \ell \mid Y, T] \propto \\ N(Y; f_n, \sigma^2 I_n)\; N(f_n, 0_n,\; t^2 \mathcal{T}_\ell)\; \text{IG}(\sigma^2; a_\sigma, a_\sigma)\; \text{IG}(t^2; a_t, a_t)\; \text{Unif}(\ell; a_\ell, b_\ell). \qquad [S9]$$

The posterior distribution is proportional to the product of the likelihood function and prior density functions. However, the evidence (the normalizing constant) is intractable. Thus, it is not feasible to directly use the joint posterior distribution for inference since the posterior distribution does not commit to a standard distribution family. Instead, we implemented the Markov Chain Monte Carlo (MCMC)[7] method to draw a set of posterior samples of size S,

$$\left\{\left(f^{(s)}(t_1), \cdots, f^{(s)}(t_n), \sigma^{2^{(s)}}, t^{2^{(s)}}, \ell^{(s)}\right), s = 1, \cdots, S\right\}.$$

We used the posterior sample mean as the estimate of the binding counts, as

$$\hat{f}(t_i) = \frac{1}{S}\sum_{s=1}^{S} f^{(s)}(t_i)\,, i = 1, \cdots, n. \qquad [S10]$$

The posterior standard deviation in estimating $\hat{f}(t_i)$ is estimated by the standard deviation of the posterior samples

$$\hat{s}_i = \sqrt{\frac{1}{S-1}\sum_{s=1}^{S}\left\{f^{(s)}(t_i) - \hat{f}(t_i)\right\}^2}\,, i = 1, \cdots, n. \qquad [S11]$$

Equations S10 and S11 are used to provide posterior estimates of binding counts with $S = 1000$ posterior samples when analyzing the real data.

Posterior computation. Markov Chain Monte Carlo (MCMC) algorithm generates Markov Chain samples iteratively, of which the distribution converges to the target (posterior) distribution as the

number of iterations goes to infinity. In practice, it is justified that the distribution of the MCMC samples approximates the posterior distributions, we consider using the Gibbs sampling technique which is one of the common MCM algorithms used for Bayesian inference[8]. The Gibbs sampler iteratively samples the unknown parameters separately from their conditional posterior distributions, forming an updating circle: 1) sample from $[f_n \mid \sigma^2, t^2, \ell]$; 2) sample from $[t^2 \mid f_n, \sigma^2, \ell]$; 3) samples from $[\sigma^2 \mid f_n, t^2, \ell]$ and 4) sample from $[\ell \mid \sigma^2, t^2, f_n]$. It is verified that the joint distribution of samples drawn from the updating circle also converges to the true posterior distribution. The detailed conditional distributions and corresponding sampling procedures are laid out as follows

1. Update $[f_n \mid \sigma^2, t^2, \ell, Y, T] \sim N(\mu_f, \Sigma_f)$ where $\Sigma_f = \left(\frac{\mathcal{T}_\ell^{-1}}{t^2} + \sigma^{-2} I_n\right)^{-1}$ and $\mu_f = \frac{\Sigma_f Y}{\sigma^2}$;
2. Update $[t^2 \mid f_n, \sigma^2, \ell, Y, T] \sim \mathrm{IG}(\tilde{a}_t, \tilde{b}_t)$ where $\tilde{a}_t = a_t + n/2$ and $\tilde{b}_t = a_t + \frac{f_n^T \mathcal{T}_\ell^{-1} f_n}{2}$. We denote by f_n^T the transpose of vector f_n.
3. Update $[\sigma^2 \mid f_n, t^2, \ell, Y, T] \sim \mathrm{IG}(\tilde{a}_\sigma, \tilde{b}_\sigma)$ where $\tilde{a}_\sigma = a_\sigma + n/2$ and $\tilde{b}_\sigma = a_\sigma + \frac{(Y-f_n)^T(Y-f_n)}{2}$.
4. Update $[\ell \mid \sigma^2, t^2, f, Y, T] \propto |\mathcal{T}_\ell|^{-1/2} \exp\left\{\frac{f_n^T \mathcal{T}_\ell^{-1} f_n}{2}\right\} \mathbb{I}_{(a_\ell, b_\ell)}(\ell)$ using Metropolis-Hasting algorithm. We write $|\mathcal{T}_\ell|$ as the determinant of matrix $\mathcal{T}_\ell$, and $\mathbb{I}_A(x)$ denotes the indicator function of x in set A, that $\mathbb{I}_A(x) = 1$ if $x \in$ A and $\mathbb{I}_A(x) = 0$ otherwise.

In fitting the real binding count data, we implemented 3000 iterations, discarded the first 2000 iterations as burn-ins, and stored the last 1000 iterations as our posterior samples. Our empirical results also indicated that the Markov chain mixed well, the details are omitted here.

Note S4. Comparison of LFSM-immunoassay at different counting times.

We studied the influence of counting time on the detection limit and the precision by comparing the standard curves of NT-proBNP detection under clinical settings for counting times of 5 and 10 mins. The first 5 mins of data in Figure 3 were processed in the same way and shown in Figure S18 (details of each replicate can be found in Figure S19). The detection limits are 5.22 pg/mL for 5 mins and 4.70 pg/mL for 10 mins. Compared with the fitting precision of 10 mins counting time, the r-square of 5 mins drops from 0.99 to 0.97 (Figure S18b). The correlation coefficient between our method and Roche's assay also drops from 0.99 to 0.95 (Figure S18c). Therefore, longer counting time within clinically acceptable range can improve the sensitivity and precision of LFSM-immunoassay by enabling more binding events to be counted[5].

Note S5. The effect of detection antibody concentration on NT-proBNP detection.

We compared NT-proBNP detection results for detection antibody concentrations of 10 nM and 50 nM. The number of binding events in the blank experiments are similar for the two

concentrations (Figure S16a). However, the response signal for the 50 nM is bigger than the 10 nM, after the sensor was incubated in human plasma with 100 fM NT-proBNP for 10 mins.

Note S6. NT-proBNP concentration in pooled human plasma.

As NT-proBNP is a stable protein and exists in the blood of both patients and healthy people, the endogenous concentration of NT-proBNP in pooled human plasma should be determined to get the accurate spiked concentration. The concentration of NT-proBNP in the human plasma pool was measured to be 8.23 pg/mL by conventional ELISA (Figure S17a). We also used our method to measure the baseline level of NT-proBNP in pooled human plasma, which was determined to be 6.71 pg/mL (Figure S17b). Based on these results, the recovery was found to be 18.5%.

Note S7. Comparison of standard curves of IL-6 detection in whole blood with/without filtering by our real-time counting algorithm.

To demonstrate the ability of our algorithm for removing the nonspecific binding events, the standard curve of IL-6 detection in whole blood without applying intensity and binding time filtering to the binding events was shown in Figure S9 (Details of all data points are plotted in Figure S10). Based on the results, the IL-6 detection in whole blood could not be realized without the filtering of our real-time counting algorithm.

References

1 Young, Gavin, Hundt, Nikolas, Cole, Daniel, Fineberg, Adam, Andrecka, Joanna, Tyler, Andrew, Olerinyova, Anna, Ansari, Ayla, Marklund, Erik G., Collier, Miranda P., Chandler, Shane A., Tkachenko, Olga, Allen, Joel, Crispin, Max, Billington, Neil, Takagi, Yasuharu, Sellers, James R., Eichmann, Cédric, Selenko, Philipp, Frey, Lukas, Riek, Roland, Galpin, Martin R., Struwe, Weston B., Benesch, Justin L. P. & Kukura, Philipp. Quantitative mass imaging of single biological macromolecules. *Science* **360**, 423-427, doi:10.1126/science.aar5839 (2018, Ref#15 of main text).

2 Yang, Lei, Parton, Richard, Ball, Graeme, Qiu, Zhen, Greenaway, Alan H., Davis, Ilan & Lu, Weiping. An adaptive non-local means filter for denoising live-cell images and improving particle detection. *Journal of Structural Biology* **172**, 233-243, doi:10.1016/j.jsb.2010.06.019 (2010, Ref#13 of main text).

3 Zhang, Pengfei, Ma, Guangzhong, Dong, Wei, Wan, Zijian, Wang, Shaopeng & Tao, Nongjian. Plasmonic scattering imaging of single proteins and binding kinetics. *Nature Methods* **17**, 1010-1017, doi:10.1038/s41592-020-0947-0 (2020, Ref#12 of main text).

4 Ma, Guangzhong, Zhang, Pengfei, Zhou, Xinyu, Wan, Zijian & Wang, Shaopeng. Label-Free Single-Molecule Pulldown for the Detection of Released Cellular Protein Complexes. *ACS Central Science* **8**, 1272-1281, doi:10.1021/acscentsci.2c00602 (2022, Ref#16 of main text).

5 Jing, Wenwen, Wang, Yan, Yang, Yunze, Wang, Yi, Ma, Guangzhong, Wang, Shaopeng & Tao, Nongjian. Time-Resolved Digital Immunoassay for Rapid and Sensitive Quantitation of Procalcitonin with Plasmonic Imaging. *ACS Nano* **13**, 8609-8617, doi:10.1021/acsnano.9b02771 (2019, Ref#11 of main text).

6 Gelman, Andrew. Prior distributions for variance parameters in hierarchical models (comment on article by Browne and Draper). *Bayesian Analysis* **1**, 515-534, 520 (2006).

7 Gamerman, Dani & Lopes, Hedibert F. *Markov chain Monte Carlo: stochastic simulation for Bayesian inference*. (Chapman and Hall/CRC, 2006).

8 Gelfand, Alan E. Gibbs sampling. *Journal of the American statistical Association* **95**, 1300-1304 (2000).
